# The M2 proteins of bat influenza A viruses reveal atypical features compared to conventional M2 proteins

**DOI:** 10.1128/jvi.00388-23

**Published:** 2023-08-04

**Authors:** Danielle Thompson, Christiana Victoria Cismaru, Jean-Sebastien Rougier, Martin Schwemmle, Gert Zimmer

**Affiliations:** 1 Institute of Virology and Immunology, Mittelhäusern, Switzerland; 2 Graduate School for Cellular and Biomedical Sciences, University of Bern, Bern, Switzerland; 3 Institute of Biochemistry and Molecular Medicine, University of Bern, Bern, Switzerland; 4 Institute of Virology, Medical Center – University of Freiburg, Freiburg im Breisgau, Germany; 5 Department of Pathology and Infectious Diseases, Vetsuisse Faculty, University of Bern, Bern, Switzerland; Emory University School of Medicine, Atlanta, Georgia, USA

**Keywords:** viroporin, protein trafficking, virus uncoating, hemagglutinin, amantadine

## Abstract

The influenza A virus (IAV) M2 protein has proton channel activity, which plays a role in virus uncoating and may help to preserve the metastable conformation of the IAV hemagglutinin (HA). In contrast to the highly conserved M2 proteins of conventional IAV, the primary sequences of bat IAV H17N10 and H18N11 M2 proteins show remarkable divergence, suggesting that these proteins may differ in their biological function. We, therefore, assessed the proton channel activity of bat IAV M2 proteins and investigated its role in virus replication. Here, we show that the M2 proteins of bat IAV did not fully protect acid-sensitive HA of classical IAV from low pH-induced conformational change, indicating low proton channel activity. Interestingly, the N31S substitution not only rendered bat IAV M2 proteins sensitive to inhibition by amantadine but also preserved the metastable conformation of acid-sensitive HA to a greater extent. In contrast, the acid-stable HA of H18N11 did not rely on such support by M2 protein. When mutant M2(N31S) protein was expressed in the context of chimeric H18N11/H5N1(6:2) encoding HA and NA of avian IAV H5N1, amantadine significantly inhibited virus entry, suggesting that ion channel activity supported virus uncoating. Finally, the cytoplasmic domain of the H18N11 M2 protein mediated rapid internalization of the protein from the plasma membrane leading to low-level expression at the cell surface. However, cell surface levels of H18N11 M2 protein were significantly enhanced in cells infected with the chimeric H18N11/H5N1(6:2) virus. The potential role of the N1 sialidase in arresting M2 internalization is discussed.

**IMPORTANCE:**

Bat IAV M2 proteins not only differ from the homologous proteins of classical IAV by their divergent primary sequence but are also unable to preserve the metastable conformation of acid-sensitive HA, indicating low proton channel activity. This unusual feature may help to avoid M2-mediated cytotoxic effects and inflammation in bats infected with H17N10 or H18N11. Unlike classical M2 proteins, bat IAV M2 proteins with the N31S substitution mediated increased protection of HA from acid-induced conformational change. This remarkable gain of function may help to understand how single point mutations can modulate proton channel activity. In addition, the cytoplasmic domain was found to be responsible for the low cell surface expression level of bat IAV M2 proteins. Given that the M2 cytoplasmic domain of conventional IAV is well known to participate in virus assembly at the plasma membrane, this atypical feature might have consequences for bat IAV budding and egress.

## INTRODUCTION

Several RNA and some DNA viruses encode for “viroporins,” which are small transmembrane proteins that oligomerize to form a hydrophilic pore that allows specific ions to pass the membrane along the concentration gradient (1, 2). The ion channel activity of viroporins plays a role in several stages of the viral life cycle, but there are also numerous functions of viroporins known that are not linked to their ion channel activity (1 - 6). The influenza A virus (IAV) matrix protein 2 (M2) is a prototype ion channel protein that has been extensively characterized (7 - 12).

The M2 protein is encoded by IAV genomic RNA segment 7, which also encodes the matrix protein 1. While the M1 protein is produced from the unspliced RNA transcript, M2 is translated from the spliced RNA. As a consequence of RNA splicing, the two proteins share the same nine amino acids (aa) at their respective N-terminus. The M2 proteins of conventional IAV are highly conserved type III membrane proteins, which consist of a short N-terminal ectodomain (ED, aa 1-25), a transmembrane domain (TM, aa 26-44) and a long cytoplasmic domain (CD, aa 45-97), which comprises an amphipathic helix (AH, aa 45-62) and a C-terminal tail (CT, aa 63-97). The M2 protein primarily localizes to the Golgi and the plasma membrane of infected cells, but some copies of the molecule are also integrated into the viral envelope (13). The tetrameric form of M2 has ion channel activity with high selectivity for protons and low selectivity for Na^+^ and K^+^ ions (8, 14, 15). The TM region of the M2 tetramer forms the ion-conducting pore. It contains a characteristic His37-X-X-X-Trp41 motif which is highly conserved in all IAV M2 proteins. His37 accounts for the proton selectivity and acid activation of the M2 channel proteins (16 - 19), while Trp41 acts as a gate that opens and closes the pore of the ion channel (7, 20, 21). Amantadine (AMT) is a small adamantane compound with antiviral activity that binds to the pore and thereby blocks ion channel activity (21). AMT-resistant IAV reveal characteristic mutations in the pore-forming domain (e.g., S31N) which abrogate binding of the drug.

The ion channel activity of M2 protein is crucial for the IAV uncoating process (22). Following internalization of IAV by receptor-mediated endocytosis, a vacuolar-type H^+^-ATPase leads to a progressive increase of the proton concentration in the endosomal lumen. The acidic milieu in the endosome activates the M2 ion channel protein embedded in the viral envelope (23). The inward proton flux into the virion promotes loosening of M1 interactions with itself and other viral components such as HA and viral ribonucleoprotein complexes (vRNP), thereby decreasing rigidity of the virus particle (23 - 25). When the pH value drops beyond a critical pH threshold level, the viral hemagglutinin (HA) undergoes a conformational change and triggers the fusion of the viral envelope with the endosomal membrane (26). As the M1 protein has already separated from the RNPs, the nuclear localization signal of the nucleoprotein (NP) can be recognized which allows the active transport of the RNPs into the nucleus, where replication and transcription of the viral genome takes place. The uncoating step is inhibited by the antiviral compounds AMT and rimantadine, which prevent protons from passing the channel (21).

For some IAV, an additional function has been assigned to the M2 protein. The ion channel protein raises the pH in the secretory pathway and thereby preserves the native conformation of HA during its transport through acidic compartments such as the Golgi apparatus (27). If the ion channel is blocked by AMT, HA is at risk of undergoing a premature conformational change, which is irreversible. As a consequence, HA is transported to the plasma membrane and integrated into the viral envelope in a fusion-incompetent form. M2 ion channel activity also affects cellular ion homeostasis and contributes to IAV pathogenesis by dysregulating protein transport and maturation in the secretory pathway (28, 29), by altering cellular ion channel functions in the airways (30 - 32), and by triggering the inflammasome (33).

In 2012 and 2013, the genomic sequences of novel influenza A-like viruses were identified in Guatemala in specimens of the little yellow-shouldered bat *Sturnira lilium* (34) and in Peru in feces of the fruit bat *Artibeus planirostris* (35). The phylogenetic analysis of the genomic RNA segments encoding the putative envelope glycoproteins HA and NA led to the classification of bat IAV into the new subtypes H17N10 and H18N11. Bat IAV differ from conventional IAVs in several fundamental aspects. The surface glycoproteins lack the canonical receptor-binding and -destroying activities of classical HA and NA, respectively (35 - 37). The bat IAV HA glycoproteins H17 and H18 do not use sialic acid residues for attachment to the host cell but rather take advantage of the MHC class II protein complex for entry into host cells of different species (38). The ectodomain of the NA-like protein seemed to be dispensable for virus replication in cell culture and in mice (39), but turned out to be important for efficient transmission in the bat natural host (40). These findings indicate that the NA-like protein has an impact on tissue tropism and transmission, although the exact biological function of this envelope glycoprotein is not known yet.

Another unusual feature of bat IAV is the incompatibility of the packaging signals of most of their RNA segments with those of conventional IAV (41, 42). Only when the packaging signals of bat IAV RNA segments four and six were used, chimeric bat IAV harboring the envelope glycoproteins of the mouse-adapted A/SC35M (H7N7) could be generated by reverse genetics (41). The chimeric virus turned out to be resistant to amantadine, in line with the presence of an asparagine residue at position 31. Interestingly, when the chimeric virus was passaged on chicken DF-1 cells or embryonated chicken eggs, the asparagine at position 31 rapidly mutated to serine, which rendered the virus sensitive to amantadine (41). The functional relevance of this adaptive mutation remains unclear since the ion channel activity of the bat IAV M2 proteins has not been studied so far.

In the present study, we analyzed the bat IAV M2 proteins with respect to subcellular localization, proton channel activity (PCA), and functional importance for virus entry and HA metastable conformation in the secretory pathway. Our findings suggest that bat IAV M2 proteins differ fundamentally in several aspects from the M2 proteins of classical IAV.

## RESULTS

### Bat IAV M2 primary sequences reveal remarkable differences compared to conventional IAV M2 proteins

**Fig 1:**
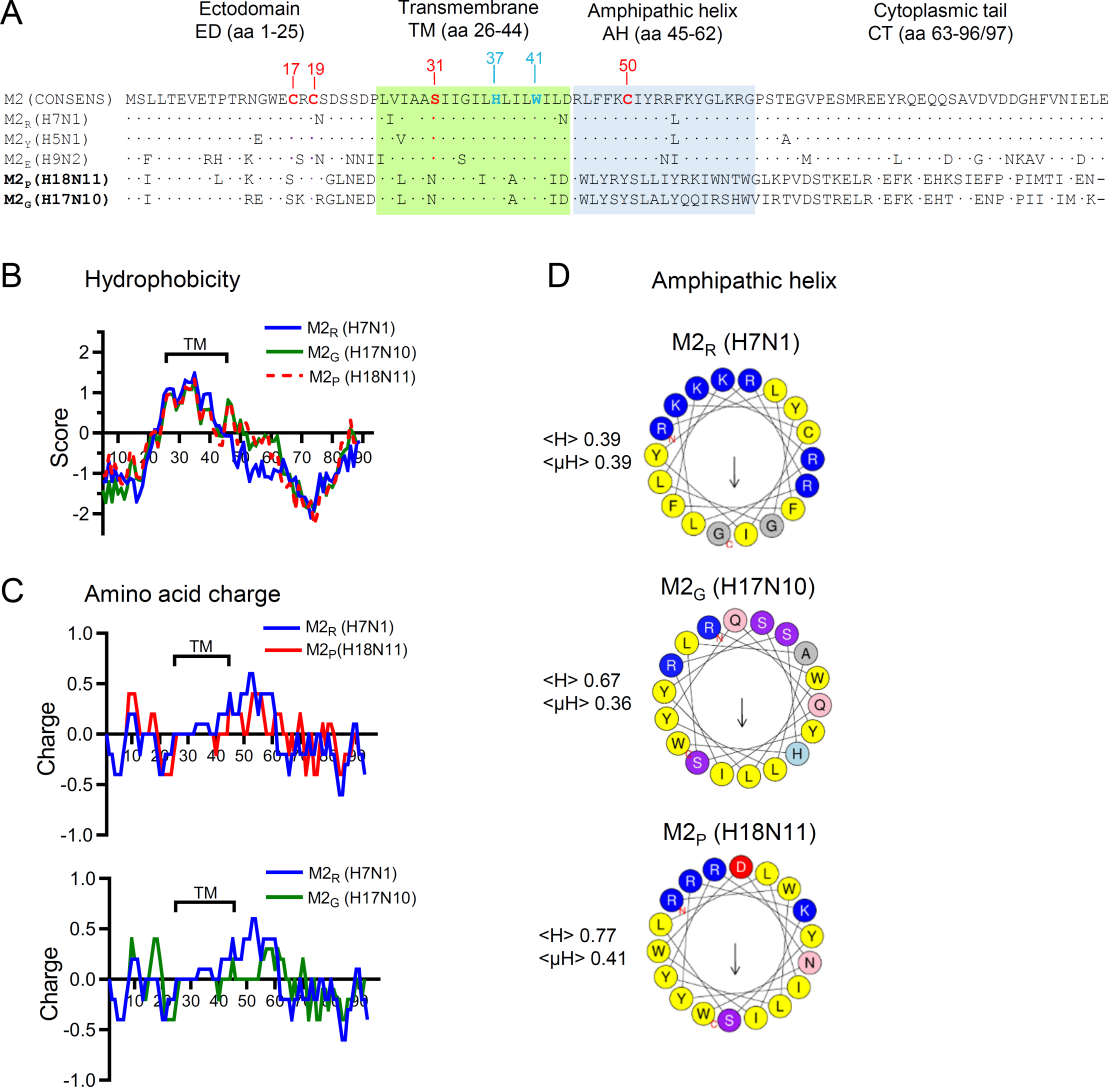
Analysis of the primary sequences of bat IAV M2 proteins. (**A**) The M2 primary sequences of A/chicken/Rostock/8/34 (H7N1), A/chicken/Yamaguchi/7/04 (H5N1), A/bat/Egypt/381OP/2017 (H9N2), A/little yellow-shouldered bat/Guatemala/164/2009 (H17N10), and A/flat-faced bat/Peru/033/2010 (H18N11) were aligned to a published M2 consensus sequence based on the primary amino acid sequences of 6413 IAV M2 proteins (41). The regions in the M2 proteins representing the actual or putative ectodomains (ED), transmembrane domains (TM), amphipathic helices (AH), and cytoplasmic tails (CT) are indicated on top of the alignment. The predicted transmembrane regions are highlighted by green and the predicted AH regions by blue backgrounds. Functionally important amino acid residues such as the conserved cysteine residues at positions 17 and 19 (disulfide bonds), amino acid residue at position 31 (susceptibility to amantadine), histidine 37 and tryptophane 41 (proton sensing and gating, respectively), and cysteine 50 (palmitoylation site) are marked by bold letters in either red or blue color. (**B, C**) Prediction of protein hydrophobicity (**B**) and protein charge (**C**). The M2 proteins of A/chicken/Rostock/8/34 (H7N1) (M2_R_), A/bat/Guatemala/164/2009 (H17N10) (M2_G_), and A/flat-faced bat/Peru/033/2010 (H18N11) (M2_P_) are plotted as blue, green, and red curves, respectively. The predicted TM domains are indicated. (**D**) Prediction of amphipathic helixes by Heliquest in the region comprising amino acids (aa) 45–62. Hydrophobic amino acids are depicted in yellow, basic amino acids in blue, acidic amino acids in red or pink, hydroxy amino acids are shown in violet, and small non-charged amino acids are highlighted in gray. The <µH> (hydrophobic moment) and <H> (hydrophobicity) indices are indicated on the left side of each wheel. The arrows point to the hydrophobic faces of the helices. Their lengths correspond to the hydrophobic moments.

The open reading frames of the bat IAV M2 proteins are composed of 96 aa, whereas the M2 proteins of conventional IAV always comprise 97 aa (Fig. 1A). For prediction of putative TM domains, we ran a hydrophobicity plot for the M2 proteins of A/little yellow-shouldered bat/Guatemala/164/2009 (H17N10) (denoted as M2_G_) and A/flat-faced bat/Peru/033/2010 (H18N11) (denoted as M2_P_) and compared them with the M2 protein of the conventional avian IAV A/FPV/chicken/Rostock/1934 (H7N1) (denoted as M2_R_) (Fig. 1B). For all three M2 proteins the hydropathy graph followed a very similar trend except for the region between aa 44 and 62, which corresponds to the AH of conventional IAV (Fig. 1B). The AH domain of M2_R_ protein revealed a higher hydrophilicity than the bat IAV M2 proteins. Nevertheless, a hydrophobic region between aa 26 and 43, which correspond to the TM domain of conventional IAV M2 proteins (7), was also detected in the bat IAV M2 protein. When a plot for charged aa residues was performed, the predicted TM domains of all three M2 proteins were flanked N-terminally by negatively charged aa and C-terminally by positively charged ones (Fig. 1C), in line with the topology of type III membrane proteins (43).

The M2 proteins of conventional IAV reveal an extremely high sequence conservation throughout the whole molecule (44). When the primary sequences of the H17N10 and H18N11 M2 proteins were aligned with an M2 consensus sequence representing 6,413 different isolates of conventional IAV (Fig. 1A), similarities but also remarkable differences were detected. While the M2 ED of conventional IAV normally contains two cysteine residues (Cys17 and Cys19), which stabilize the M2 tetramer by forming intermolecular disulfide bonds (45), only Cys19 was present in the bat IAV M2 proteins. Similar to conventional IAV, the TM domain of bat IAV M2 proteins harbors the His37-x-x-x-Trp41 motif, suggesting that the bat IAV M2 proteins may have PCA. In conventional IAV M2 proteins, a serine is normally found at amino acid position 31, while in AMT-resistant strains this residue is frequently mutated to asparagine (46, 47). Surprisingly, the TM domains of bat IAV M2 proteins contain Asn31 which was found to confer resistance of bat IAV to this drug (41). Using the HeliQuest algorithm (48), amphipathic helices were predicted for M2_G_ (H17N10) and M2_P_ (H18N11) with hydrophobic moments (<µH>) similar to that of M2_R_ (Fig. 1D). The AH domain of conventional IAV contains a conserved Cys residue at position 50 which is subject to post-translational palmitoylation (49, 50). This cysteine residue is absent in the CT domains of bat IAV M2 proteins. Finally, the CT domains of the bat IAV M2 proteins, including the M1 interaction site (aa 71-76) (51, 52), did not show significant homology with the corresponding region of conventional IAV. The bat IAV M2 proteins also lack the LIR motif that was shown to mediate recruitment of the autophagy protein LC3 to the plasma membrane (53).

### The H18N11 M2 protein assembles into homo-tetramers

**Fig 2:**
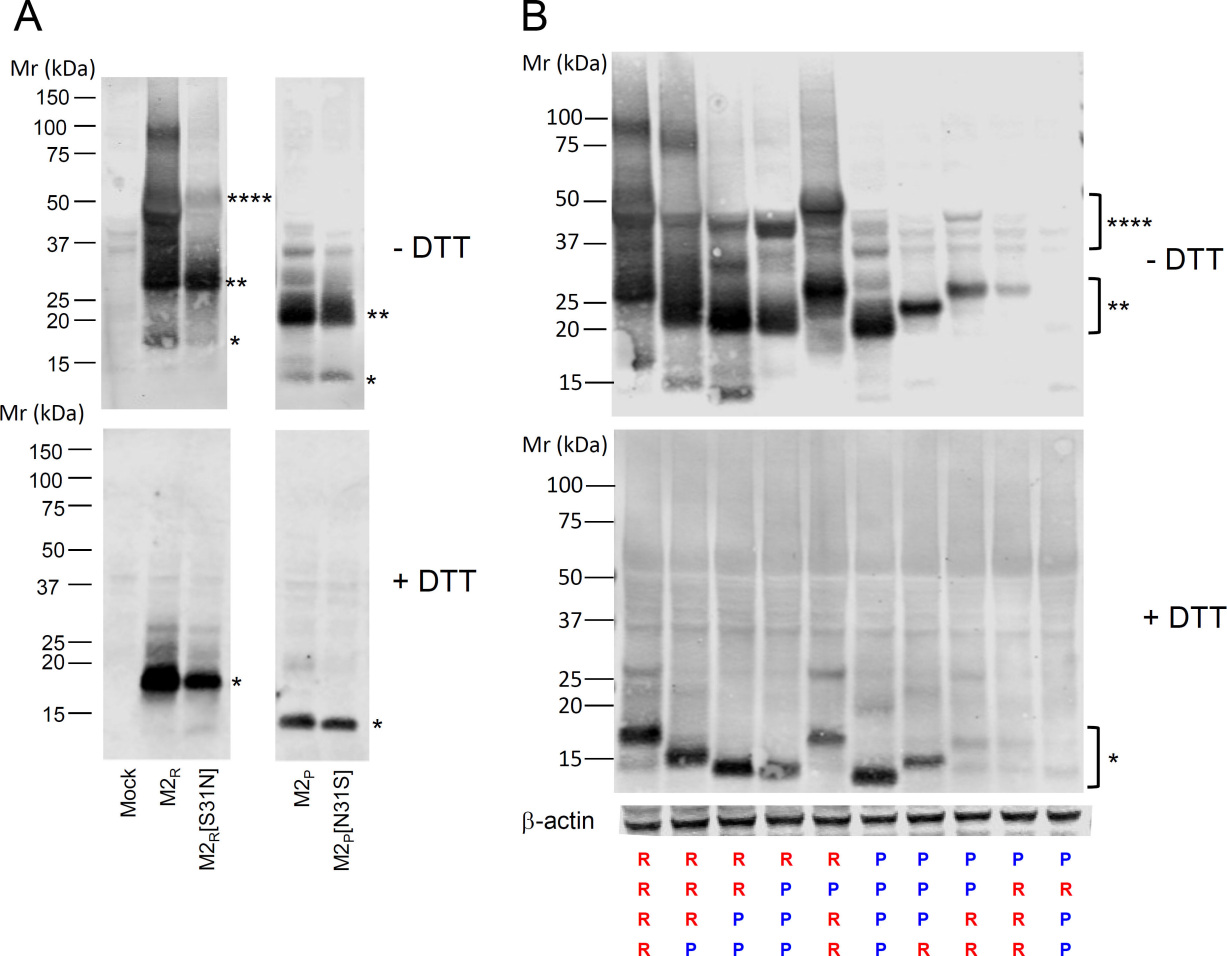
Western blot analysis of wild type and mutant M2 proteins. BHK-21 cells were transfected with expression plasmids encoding the indicated M2 proteins with an [HA] epitope at the N-terminus. At 24 hours post-transfection, the cells were lysed and proteins separated by SDS-PAGE (4%–12% gradient gel), either under non-reducing (−DTT) or reducing conditions (+DTT). The proteins were blotted onto nitrocellulose membranes and detected using a monoclonal antibody directed to the [HA] epitope. The positions of molecular weight markers are depicted, as well as the positions of monomeric (*) dimeric (**) and tetrameric (****) M2 proteins. (**A**) Western blot analysis of cells transfected with plasmids encoding either M2R, M2R(S31N), M2P, or M2P(N31S). (**B**) Western blot analysis of chimeric M2 proteins combining the ectodomains (ED), transmembrane domains (TM), amphipathic helices (AH), and cytoplasmic tails (CT) of the M2 proteins of A/chicken/Rostock/8/34 (H7N1) (denoted by R) and A/flat-faced bat/Peru/033/2010 (H18N11) (denoted by *P*).

To analyze the expression and subcellular localization of bat and conventional IAV M2 proteins, a plasmid-based system was employed. An [HA] peptide tag (YPYDVPDYA) was genetically fused to the M2 N-termini to allow detection of the different proteins with the same antibody. BHK-21 cells were transfected with the ^[HA]^M2 expression plasmids and lysed 24 hours post-transfection (p.t.). The cell lysates were separated by SDS-PAGE under non-reducing (− DTT) or reducing conditions (+ DTT) and analyzed by Western blot using an [HA]-specific monoclonal antibody (mAb). Following separation under non-reducing conditions, proteins bands migrating according to 17, 32, and 55 kDa were detected in lysates of ^[HA]^M2(H7N1)-transfected cells which likely represent the monomeric, dimeric, and tetrameric forms of the protein, respectively (Fig. 2A). Only the 17 kDa band was detected when the gel was run under reducing conditions, indicating that the oligomeric forms of mature ^[HA]^M2_R_ are normally stabilized by intermolecular disulfide bonds. The mutation S31N did not affect ^[HA]^M2_R_ oligomerization but reduced band intensity, most likely because this mutation affected M2 protein stability. The ^[HA]^M2_P_ protein of bat IAV H18N11 appeared as multiple protein species under non-reducing conditions with the most prominent one corresponding to approximately 24 kDa (Fig. 2A). Under reducing conditions, only the 12 kDa form was detected suggesting that this band represents the monomeric ^[HA]^M2_P_ and the 24 kDa band the dimeric form of the protein.

The primary sequence of ^[HA]^M2_P_ and ^[HA]^M2_R_ have similar predicted molecular masses of 12.6 and 12.4 kDa, respectively. Nevertheless, the apparent molecular masses of the two proteins differed significantly. While the ^[HA]^M2_P_ monomer appeared as a 12 kDa band, which corresponded to its predicted molecular mass, the M2_R_ monomer migrated as a 17 kDa band (Fig. 2A, + DTT). To figure out which domain would be responsible for this discrepancy, expression plasmids were generated encoding chimeric M2 proteins in which the ED, TM, AH, and CT domains of M2_P_ (each domain labeled by *P*) and M2_R_ (each domain denoted by R) were shuffled (Fig. 2B). Western blot analysis of transfected cell lysates showed that the chimeric M2(RRR-P) protein containing the CT domain of M2_P_ migrated significantly faster than M2_R_ and this shift was even more pronounced with the chimeric M2(RR-PP) protein (Fig. 2B). In contrast, the TM and ED domains had no significant effect on migration of the M2 proteins [compare M2(RR-PP) with M2(R-PPP), M2(PPPP) with M2(R-PPP), and M2(RRRR) with M2(R-P-RR)]. Vice versa, the M2(PPP-R) protein migrated slower than M2(PPPP) but faster than M2(PP-RR), while M2(P-RRR) showed the same apparent molecular mass than M2(PP-RR). The M2(PPP-R), M2(PP-RR), M2(P-RRR) proteins and in particular the M2(P-R-PP) protein showed lower band intensities compared to the parental M2(PPPP), suggesting that these chimeric proteins might be less stable. Collectively, these data suggest that determinants in the long cytoplasmic domain were responsible for the differential migration of the M2 proteins.

### The M2_P_ (H18N11) protein is expressed at the cell surface at low levels

**Fig 3:**
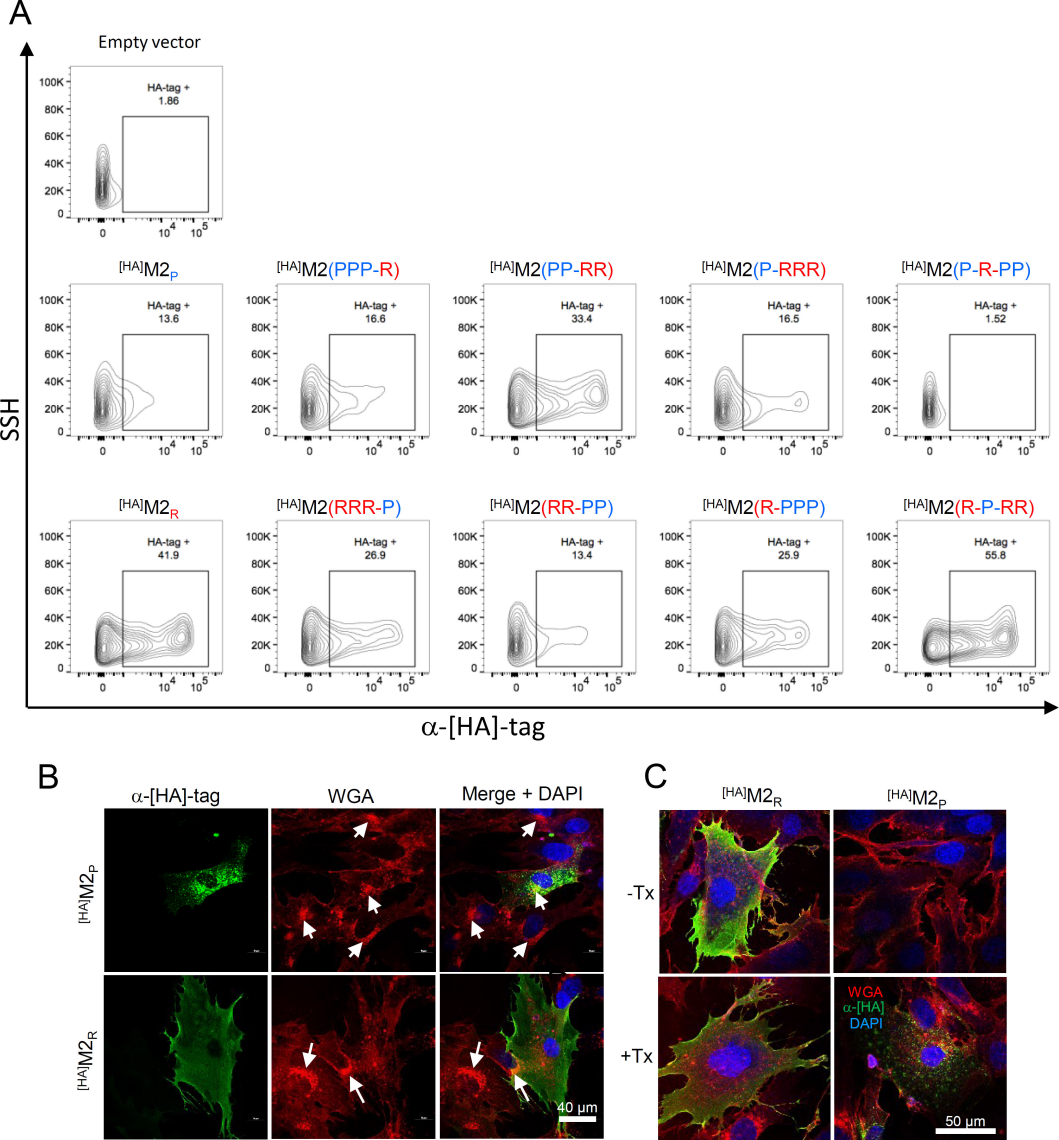
Cell surface expression of M2 proteins. (**A**) BHK-21 cells were transfected with plasmids driving the expression of the indicated [HA] epitope-tagged M2 proteins. Cells transfected with empty vector plasmid served as a negative control. At 20 hours post-transfection, the cells were stained at 4°C with an [HA] epitope-specific monoclonal antibody, subsequently with anti-mouse IgG conjugated with AlexaFluor 647, and finally analyzed by flow cytometry. The population of ^[HA]^M2-positive cells is delimitated by a rectangle in which the percent positive value is included. Individual cells were visualized using a contour plot with the y-axis representing the side-scatter height (SSH) and the x-axis standing for AlexaFluor 647 fluorescence intensity. (**B and C**) BHK-21 cells transfected with plasmids driving the expression of either ^[HA]^M2_R_ (H7N1) or ^[HA]^M2_P_ (H18N11). (**B**) At 24 hours post-transfection, the native cells were fixed with formalin, permeabilized with 0.25% of TX-100, and incubated with WGA-rhodamine (red fluorescence) and [HA]-specific mAb. The antigen-bound primary antibody was detected by an anti-mouse IgG antibody conjugated with AlexaFluor 488 (green fluorescence). Nuclei were stained with DAPI (blue fluorescence). Bar size = 40 µm. (**C**) At 24 hours post-transfection, the cells were incubated for 1 hour at 37°C with the [HA]-specific mAb, washed, and fixed with formalin. The fixed cells were either left non-permeabilized (-Tx) or were permeabilized with Triton X-100 (+Tx) prior to staining with anti-mouse AlexaFluor 488 and WGA-rhodamine. Nuclei were stained with DAPI. Bar size = 50 µm.

The IAV M2 protein plays an active role in virus budding and release (44). Furthermore, M2 is incorporated into the viral envelope and participates in virus uncoating (22, 23, 25, 54). For all these functions it is necessary that the M2 protein is transported to the cell surface where the budding process takes place. To analyze cell surface expression of M2 proteins, BHK-21 cells were transfected with expression plasmids encoding [HA]-tagged M2 proteins and analyzed at 20 hours p.t. by flow cytometry using a mAb specifically recognizing the [HA]-tag. Surprisingly, only 13.6% of the ^[HA]^M2_P_ transfected cells stained positive for the [HA] epitope compared to 1.9% positive cells following transfection with the parental plasmid and 41.9% of positive cells following transfection with ^[HA]^M2_R_ (Fig. 3A). To identify the protein domains that are important for cell surface transport, cells transfected with chimeric M2 proteins harboring domains from both ^[HA]^M2_P_ and ^[HA]^M2_R_ were analyzed as well. The chimeric M2 protein, ^[HA]^M2(PPP-R), showed only a slightly enhanced cell surface expression compared to M2_P_, while a much stronger cell surface staining was observed with the ^[HA]^M2(PP-RR) protein. The ^[HA]^M2(P-RRR) protein also revealed enhanced cell surface expression, although the increase was less pronounced compared to ^[HA]^M2(PP-RR). In contrast, ^[HA]^M2(P-R-PP), a chimeric ^[HA]^M2_P_ protein harboring the M2_R_ TM domain, showed lower cell surface expression compared to ^[HA]^M2_P_, indicating that this domain had a negative rather than a positive effect on cell surface transport. Interestingly, the AH and CT domains of ^[HA]^M2_P_ had a negative impact on the cell surface expression levels of ^[HA]^M2(RRR-P) and ^[HA]^M2(RR-PP) compared to parental ^[HA]^M2_R_ protein (Fig. 3A). The ^[HA]^M2(R-PPP) also showed reduced expression levels although not as pronounced as the ^[HA]^M2(RR-PP) protein. In contrast, ^[HA]^M2(R-P-RR) protein showed no reduced cell surface staining indicating that the TM domain of ^[HA]^M2_P_ had no negative effect on the cell surface expression of this chimeric protein.

Intracellular staining of the transfected cells with the [HA] epitope-specific antibody revealed that the median fluorescence intensity (MFI) for ^[HA]^M2_P_ is much lower compared to the MFI detected for ^[HA]^M2_R_ (Fig. S1), suggesting that ^[HA]^M2_P_ was less stable. By swapping the AH and CT domains with those from ^[HA]^M2_R_, the total expression levels increased, suggesting that these domains had a stabilizing effect on the protein. When the AH and CT domains of ^[HA]^M2_R_ were replaced by those from ^[HA]^M2_P_, the overall expression levels stayed the same, but the percentage of ^[HA]^M2(RR-PP) at the cell surface declined. Compared to the parental ^[HA]^M2_R_ protein, ^[HA]^M2(R-P-RR) protein revealed a slightly reduced overall expression level, while the relative cell surface expression level was even higher. In contrast, the ^[HA]^M2(P-R-PP) protein showed a reduced relative cell surface expression compared to the parental ^[HA]^M2_P_ protein. Collectively, these findings suggest that the CT and AH domains have an impact on ^[HA]^M2 protein stability and/or subcellular localization.

To analyze subcellular localization of M2 proteins, BHK-21 cells were transfected with expression plasmids encoding [HA]-tagged M2 proteins. The cells were fixed with formalin at 24 hours p.t., permeabilized with Triton-X100, and analyzed by indirect immunofluorescence. Cells transfected with expression plasmid encoding ^[HA]^M2_P_ protein showed a punctate signal for the [HA] epitope in the cytosol, while the plasma membrane was not labeled with the antibody (Fig. 3B, upper panel). In contrast, a strong labeling of the plasma membrane was observed for ^[HA]^M2_R_ (Fig. 3B, lower panel), indicating that this M2 protein accumulates at the cell surface. The transfected cells were also labeled with a rhodamine conjugate of wheat germ agglutinin (WGA), a lectin that binds to sialic acid and N-acetylglucosaminyl residues and labels cellular compartments in which glycoconjugates are highly abundant such as the Golgi apparatus (55). Interestingly, in cells expressing ^[HA]^M2_R_ the Golgi apparatus appeared dispersed (Fig. 3B, lower panel), a phenomenon that has been linked to high M2 proton channel activity in the Golgi membrane (56). In contrast, the Golgi apparatus seemed to be intact in cells expressing ^[HA]^M2_P_ (Fig. 3B, upper panel). We hypothesized that ^[HA]^M2_P_ is transported to the plasma membrane but does not stay there because it is rapidly internalized. To test this hypothesis, we incubated the ^[HA]^M2_P_ transfected cells with the [HA]-specific antibody for 1 hour at 37°C prior to fixation with formalin. When the non-permeabilized cells were subsequently incubated with the Alexa Fluor 488-labeled secondary antibody, none or very faint specific labeling of the cells was observed (Fig. 3C, right hand panel). However, when the fixed cells were permeabilized with Triton-X-100 and then incubated with the secondary antibody, several punctate vesicle-like structures were labeled, indicating that the primary anti-[HA]tag antibody was internalized when incubated with the cells at 37°C. In contrast, ^[HA]^M2_R_ was labeled with the [HA]tag antibody at the cell surface, irrespective of the permeabilization of the fixed cells (Fig. 3C, left hand panel).

### Bat IAV M2 proteins do not preserve the metastable conformation of acid-sensitive HA

**Fig 4:**
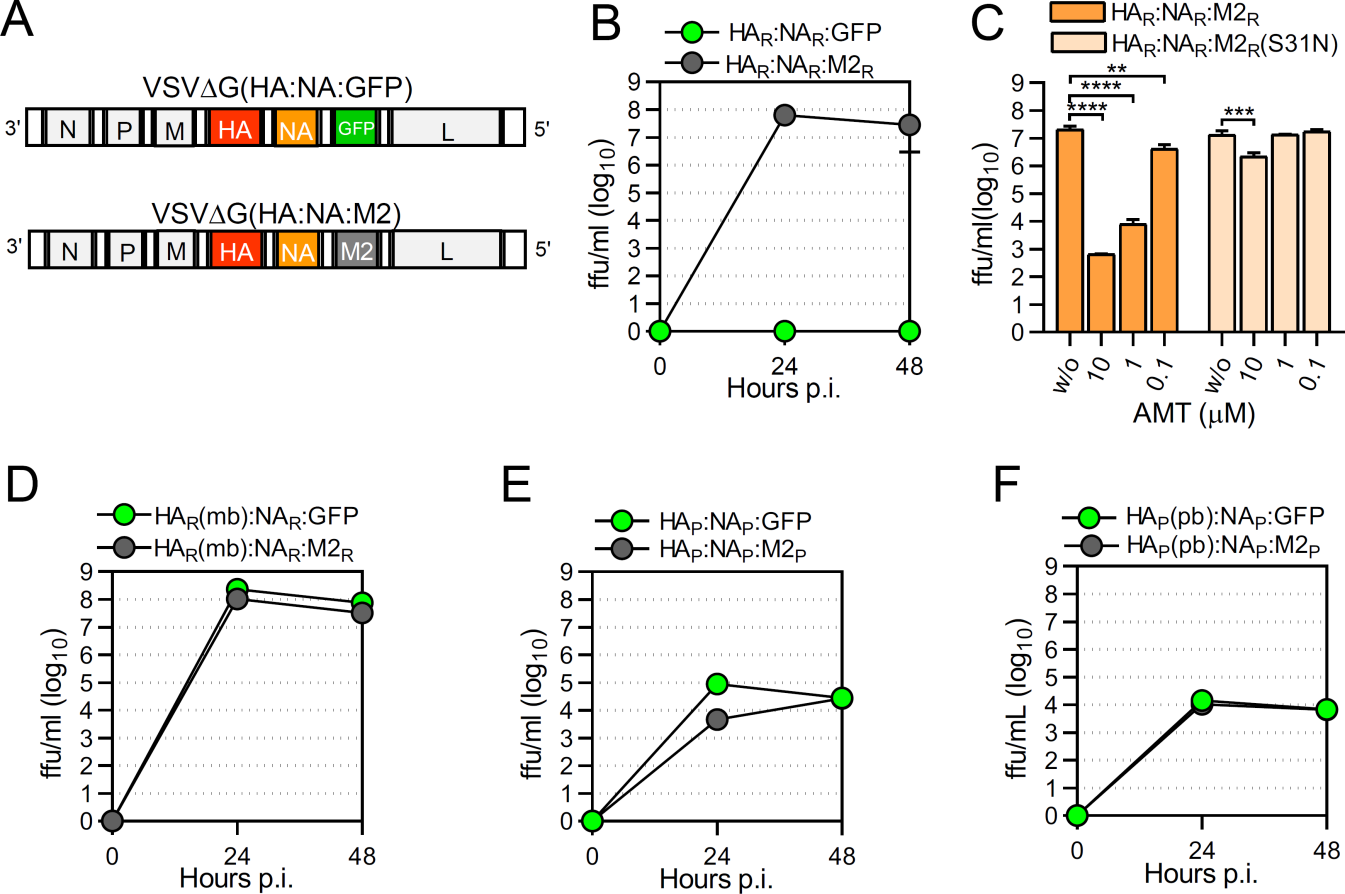
Importance of M2 protein for the replication of chimeric VSVΔG(HA:NA:M2) vectors. (**A**) Genome schemata of chimeric VSV encoding HA and NA along with either M2 or GFP protein. (**B**) Replication kinetics of chimeric VSV∆G(HA_R_:NA_R_:GFP) (green symbols) and VSV∆G(HA_R_:NA_R_:M2_R_) (gray symbols) on MDCK-II cells. The viruses were *trans*-complemented with the VSV envelope G protein by propagation on helper cells conditionally expressing VSV G protein. The cells were infected using an MOI of 0.01 ffu/cell and virus released into the cell culture supernatant was collected at the indicated times and titrated on MDCK-II cells. The lower limit of detection was 25 ffu/mL. (**C**) Effect of amantadine on the release of VSVΔG(HA_R_:NA_R_:M2_R_) and VSVΔG(HA_R_:NA_R_:M2_R_[S31N]). MDCK-II cells were infected with the indicated recombinant VSV (MOI of 1 ffu/cell) and maintained in the presence of amantadine at the indicated concentrations. Infectious virus released into the cell culture supernatant was collected at the indicated time points and infectious virus titers determined. Mean values and SD of three infection experiments are shown. Statistical analysis was performed using the one-way ANOVA test. **P < 0,005; ***P < 0.001; ****P < 0.0001. (**D**) Replication kinetics of chimeric VSV∆G(HA_R_(mb):NA_R_:GFP) (green symbols) and VSV∆G(HA_R_(mb):NA_R_:M2_R_) (gray symbols) on MDCK-II cells. The replication kinetics was performed in the presence of trypsin to account for the mb proteolytic cleavage site of HA_R_(mb). (**E**) Replication kinetics of VSVΔG(HA_P_:NA_P_:GFP) and VSVΔG(HA_P_:NA_P_:M2_P_) on MDCK-II cells (m.o.i. = 0.01 ffu/cell) in the absence of trypsin. (**F**) Replication kinetics of VSVΔG(HA_P_(pb):NA_P_:GFP) and VSVΔG(HA_P_(pb):NA_P_:M2_P_ on MDCK-II cells (m.o.i. = 0.01 ffu/cell) in the absence of trypsin. Cell culture supernatant was collected at the indicated times p.i. and infectious virus titers were determined on MDCK-II cells. (**B, D–F**) Mean values and standard deviations (SDs) of three infection experiments are shown.

Previous work has demonstrated that glycoprotein (G)-deficient vesicular stomatitis viruses (VSV) expressing the envelope glycoproteins HA and NA of the highly pathogenic avian influenza viruses (HPAIV) A/chicken/Yamaguchi/7/2004 (H5N1) and A/turkey/Italy/4580/1999 (H7N1), respectively, were able to replicate *in vitro* in an autonomous manner (57). By generating viruses with a similar gene arrangement, we show that the chimeric virus VSV∆G(HA_R_:NA_R_:GFP), which encodes for the HA_R_ and NA_R_ envelope glycoproteins of A/FPV/chicken/Rostock/1934 (H7N1) (Fig. 4A), did not replicate autonomously on MDCK-II cells (Fig. 4B). In contrast, VSV∆G(HA_R_:NA_R_:M2_R_), which additionally encodes the M2_R_ protein (Fig. 4A), replicated efficiently on MDCK-II cells reaching maximum titers of almost 10^7^ f.f.u./mL at 48 hours post-infection (p.i.) (Fig. 4B). To figure out whether PCA of M2_R_ conferred infectivity to VSVΔG(HA_R_:NA_R_:M2_R_), we infected MDCK-II cells using a multiplicity of infection (m.o.i.) of 1 f.f.u./cell and subsequently incubated the cells with AMT. At 24 hours p.i., the number of infectious viruses released into the cell culture supernatant was determined (Fig. 4C). Infectious titers of VSVΔG(HA_R_:NA_R_:M2_R_) dropped by 4.5 log_10_ when the cells were treated with 10 µM of AMT and by 3.4 log_10_ and 0.7 log_10_ when the cells were incubated with 1 µM and 0.1 µM of AMT, respectively, demonstrating that VSVΔG(HA_R_:NA_R_:M2_R_) was inhibited by AMT in a dose-dependent manner. In contrast, VSVΔG(HA_R_:NA_R_:M2_R_[S31N]) expressing the S31N mutant M2_R_ protein, was highly resistant to inhibition by AMT (Fig. 4C). VSV∆G(HA_R_(mb):NA_R_:GFP), a chimeric virus encoding a modified HA_R_ with a monobasic (mb) proteolytic cleavage site replicated independently of M2 (Fig. 4D), indicating that proteolytic activation in the secretory pathway primes HA_R_ for pH-dependent conformational change. These findings are in line with previous reports which demonstrated that the HA of the highly pathogenic avian influenza virus A/FPV/chicken/Rostock/34 (H7N1) is particularly sensitive to low pH-triggered conformational change in the secretory pathway. In order to stay in its biologically active metastable conformation, the HA of this virus strain highly depends on M2 proton channel activity (27, 58 - 62). As the infectivity of VSVΔG(HA_R_:NA_R_:M2) relies on the incorporation of biologically active, fusion-competent HA into the viral envelope, we regard this virus as a suitable biological probe to indirectly measure M2 proton channel activity. When we refer to M2 PCA in the following text, the capacity of the M2 protein to preserve the metastable conformation of acid-sensitive HA is implied.

VSVΔG(HA_P_:NA_P_:GFP) encoding the HA_P_ and NA_P_ proteins of A/flat-faced bat/Peru/033/2010 (H18N11) has recently been shown to replicate on MDCK-II cells in the presence of trypsin (63). We compared the replication kinetics of VSVΔG(HA_P_:NA_P_:GFP) with that of VSVΔG(HA_P_:NA_P_:M2_P_), which additionally encoded for the M2_P_ protein, but did not notice any enhanced virus replication in MDCK-II cells in the presence of M2_P_ (Fig. 4E). Since HA_P_ (H18N11) contains a monobasic proteolytic cleavage site, it may not be primed for a pH-dependent conformational change and thus may not rely on M2 PCA. We, therefore, generated two additional chimeric viruses, VSVΔG(HA_P_(pb):NA_P_:GFP) and VSVΔG(HA_P_(pb):NA_P_:M2_P_), both encoding a modified HA_P_ protein with a polybasic (bp) proteolytic cleavage site (63). Both viruses replicated independently of trypsin on MDCK-II cells but showed no significant difference in replication kinetics (Fig. 4F), suggesting that the HA_P_ protein does not require help from the M2_P_ protein to maintain its metastable conformation.

### Bat IAV M2 proteins imperfectly rescue the metastable conformation of acid-sensitive HA

**Fig 5:**
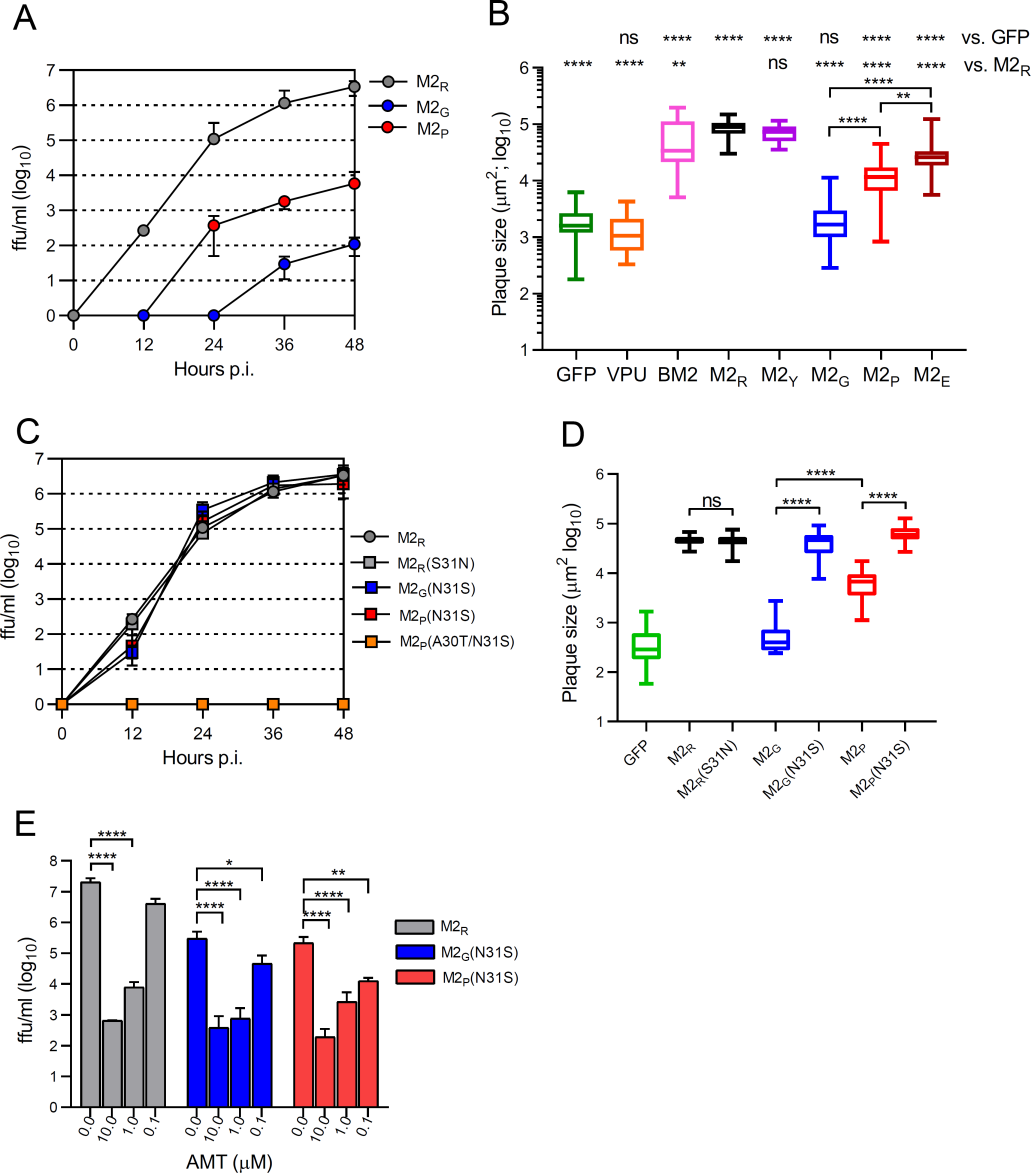
Analysis of the capacity of bat IAV M2 proteins to preserve the metastable conformation of acid-sensitive HA. (**A, C**) Replication kinetics of VSVΔG(HA_R_:NA_R_:M2) encoding the acid-labile HA protein of A/chicken/Rostock/8/1934 (H7N1). HEK 293T cells were inoculated (MOI of 0.0001 ffu/cell) with chimeric VSVΔG(HA_R_:NA_R_:M2) encoding the indicated M2 proteins. Cell culture supernatant was collected at the indicated times p.i. and infectious virus titers were determined by titration on BHK-21 cells. The assay limit of detection was 25 ffu/mL. (**B, D**) Plaque size assays were performed by infecting BHK-21 cell monolayers with either VSVΔG(HA_R_:NA_R_:GFP) or VSVΔG(HA_R_:NA_R_:viroporin) encoding one of the indicated wild type or mutant viroporins: HIV-1 vpu protein, influenza B virus BM2 protein, M2_R_ protein of A/chicken/Rostock/8/1934 (H7N1), M2_Y_ of A/chicken/Yamaguchi/7/2004 (H5N1), M2_G_ of A/bat/Guatemala/164/2009 (H17N10), M2_P_ of A/flat-faced bat/Peru/033/2010 (H18N11), and M2_E_ of A/bat/Egypt/381OP/2017 (H9N2). The viruses were allowed to spread in the monolayer covered with a methylcellulose overlay medium for 30 hours before fixation with formalin and immunolabeling of the infected cells with a VSV M protein-specific mAb. Box & Whiskers (min to max) plots of at least 20 plaques per virus are depicted. (**E**) Sensitivity of chimeric VSV to inhibition to amantadine. HEK 293T cells were infected with the indicated recombinant VSV (MOI = 1 ffu/cell) and maintained in the presence of amantadine at the indicated concentrations. At 24 hours p.i., the infectious virus released into the cell culture supernatant was titrated on BHK-21 cells. Mean values and SD of three infection experiments are shown. (**B, D, E**) Significant differences were assessed by the one-way ANOVA test with Dunnett`s multiple comparisons tests (ns, not significant; *P < 0.05; **P < 0.01; ***P < 0.001; ****P < 0.0001).

To assess the PCA of bat IAV M2 proteins, we replaced the M2_R_ gene in VSVΔG(HA_R_:NA_R_:M2_R_) with either M2_G_ (H17N10) or M2_P_ (H18N11) and performed multicycle replication kinetics on HEK 293T cells. Compared to VSVΔG(HA_R_:NA_R_:M2_R_), the chimeric viruses replicated only inefficiently, suggesting that the bat IAV M2 proteins have lower PCA than conventional IAV M2 proteins (Fig. 5A). Furthermore, VSVΔG(HA_R_:NA_R_:M2_G_) replicated less efficiently than VSVΔG(HA_R_:NA_R_:M2_P_), suggesting that the M2_P_ protein has higher PCA than the M2_G_ protein. The impact of M2 proteins on chimeric VSV replication was also analyzed by a plaque size assay on BHK-21 cells (Fig. 5B), which was found to correlate with virus titers (Fig. S2). While VSVΔG(HA_R_:NA_R_:M2_R_) produced large plaques in the cell monolayer, the infection with VSVΔG(HA_R_:NA_R_:GFP) remained localized to the primary infected cell. Plaques formed by VSVΔG(HA_R_:NA_R_:M2_G_) were not significantly larger than those formed by VSVΔG(HA_R_:NA_R_:GFP), indicating very low PCA. Infection with VSVΔG(HA_R_:NA_R_:M2_P_) resulted in plaques that were significantly larger than those formed by VSVΔG(HA_R_:NA_R_:M2_G_) but still smaller than those induced by VSVΔG(HA_R_:NA_R_:M2_R_). We also analyzed chimeric VSV which encoded the ion channel proteins of other viruses. Chimeric VSV encoding the BM2 protein of influenza B virus (B/Lee/40) or the M2 protein of A/Yamaguchi/7/2004 (H5N1) formed plaques that were as large as those formed by VSVΔG(HA_R_:NA_R_:M2_R_). However, the M2 protein of A/bat/Egypt/381OP/2017 (H9N2) produced plaques that were smaller than those formed by VSVΔG(HA_R_:NA_R_:M2_R_) but still larger than those produced by VSVΔG(HA_R_:NA_R_:M2_G_). These findings suggest that M2 proteins derived from bat IAV may exhibit reduced proton channel activity compared to M2 proteins of conventional IAV.

### N31S mutant bat IAV M2 proteins augment replication of chimeric VSV

Our previous work with recombinant IAV containing six genomic RNA segments of bat IAV H17N10 and 2 chimeric genomic segments encoding HA and NA of a classical IAV (H7N7) revealed that Asn31 of bat M2 protein rapidly mutates to a serine or histidine residue when the virus was grown in avian DF-1 cells or embryonated chicken eggs (41). We hypothesized that the H7N7 HA, which contains a multibasic cleavage site, might have required an M2 protein with high PCA to stabilize its metastable conformation in the secretory pathway. To test this hypothesis, we introduced the N31S mutation into the bat IAV M2 proteins and generated chimeric VSV. Both VSVΔG(HA_R_:NA_R_:M2_G_[N31S]) and VSVΔG(HA_R_:NA_R_:M2_P_[N31S]) replicated on HEK 293T cells as efficiently as VSVΔG(HA_R_:NA_R_:M2_R_) (Fig. 5C), indicating that the N31S substitution had a dramatic effect on the PCA of bat IAV M2 proteins. In contrast, VSVΔG(HA_R_:NA_R_:M2_R_[S31N]) replicated as efficiently as the parental virus, indicating that the S31N mutation did not significantly affect PCA of M2_R_. The impact of M2 PCA on the replication of chimeric VSV was also evident from a plaque size assay (Fig. 5D; Fig. S2). Infection with VSVΔG(HA_R_:NA_R_:M2_R_[N31S]) or VSVΔG(HA_R_:NA_R_:M2_P_[N31S]) resulted in plaques with sizes comparable to that formed by VSVΔG(HA_R_:NA_R_:M2_R_). Finally, we tested whether AMT has antiviral activity against VSVΔG(HA_R_:NA_R_:M2_G_[N31S]) and VSVΔG(HA_R_:NA_R_:M2_P_[N31S]) and found that both viruses were inhibited by the drug in a concentration-dependent manner (Fig. 5E). Collectively, these results indicate that the N31S mutation significantly increased the PCA of bat IAV M2 proteins, whereas the capacity of conventional M2_R_ protein to support replication of VSVΔG(HA_R_:NA_R_:M2_R_) stayed similarly high, irrespective of a serine or asparagine at position 31.

### The AH and CT domains do not affect PCA of chimeric M2 proteins

**Fig 6:**
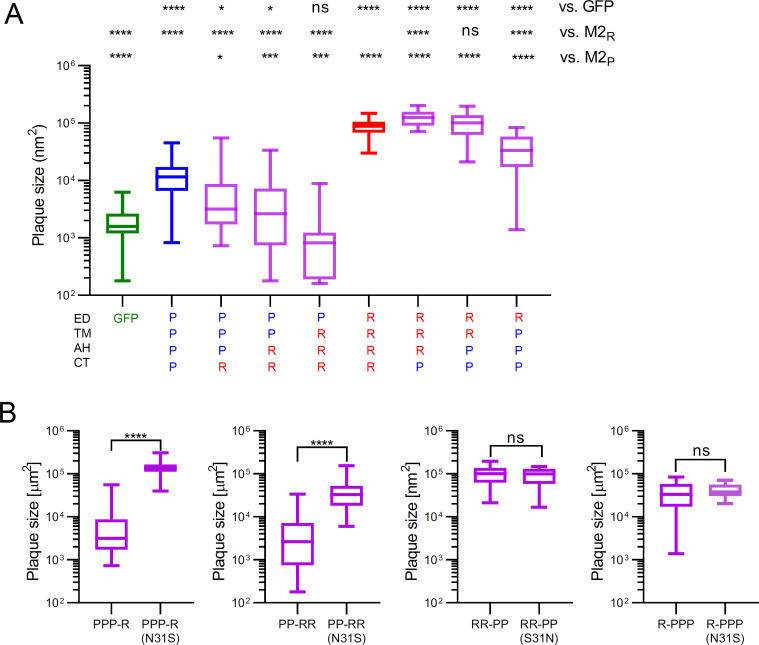
Analysis of chimeric M2 proteins on plaque size formation by chimeric VSV encoding acid-sensitive HA. (**A and B**) BHK-21 cells were infected with chimeric VSVΔG(HA_R_:NA_R_:X) where X denotes the chimeric M2 with the domain structure indicated. The cells were maintained for 30 hours at 37°C with medium containing 1% methylcellulose, fixed with formalin, and immunostained with a monoclonal antibody directed to the VSV M protein. Box & Whiskers (min to max) plots of at least 20 plaques per virus are shown. (**A**) Plaque sizes were compared by the one-way ANOVA with Dunnett`s multiple comparisons test (ns, not significant; *P < 0.05; **P < 0.01; ***P < 0.001; and ****P < 0.0001). *P* values as calculated by comparison with the indicated references are indicated on top of the plots. (**B**) Statistically significant differences as determined by the two-tailed paired Student´s t test are indicated.

Our analysis of the expression levels of chimeric M2 proteins suggested that the CT and AH domains have a strong impact on stability and cellular targeting of the proteins (see Fig. 3). To study the role of these domains in PCA, we analyzed VSVΔG(HA_R_:NA_R_:X) encoding chimeric M2 genes by plaque size assay (Fig. 6A). Viruses encoding hybrid M2 proteins with the domain architecture PPP-R, PP-RR, or P-RRR formed smaller plaques compared to M2_P_, indicating that these chimeric M2 proteins exhibited lower PCA than M2_P_, although their stability was generally higher (PPP-R, PP-RR) or at least equally high compared to M2_P_ (see Fig. 3A; Fig. S1). Virus encoding hybrid M2 protein with the domain architecture RRR-P or RR-PP formed plaques of similar size compared to M2_R_, indicating that the CT as well as the AH domain of M2_P_ did not affect PCA, although they affected M2 cell surface expression levels (see Fig. 3A). Finally, when the M2 domain architecture was changed to R-PPP, the chimeric M2 was associated with a mid-range plaque size which was in between the plaque size linked to the chimeric viruses encoding M2_R_ and M2_P_. This finding suggests that the PCA of M2_P_ is enhanced if the ectodomain is replaced by the one of M2_R_. In contrast to PPP-R and PP-RR, for which the N31S substitution caused a dramatic increase of plaque size, the N31S mutation did not affect plaque formation by chimeric VSVΔG(HA_R_:NA_R_:X) encoding the M2(R-PPP) protein (Fig. 6B). Collectively, the analysis of chimeric M2 PCA suggested that the CT and AH domains affect protein stability and subcellular targeting but have a minor effect on proton channel activity.

### Stabilization of the bat IAV M2 tetramer does not increase PCA

**Fig 7:**
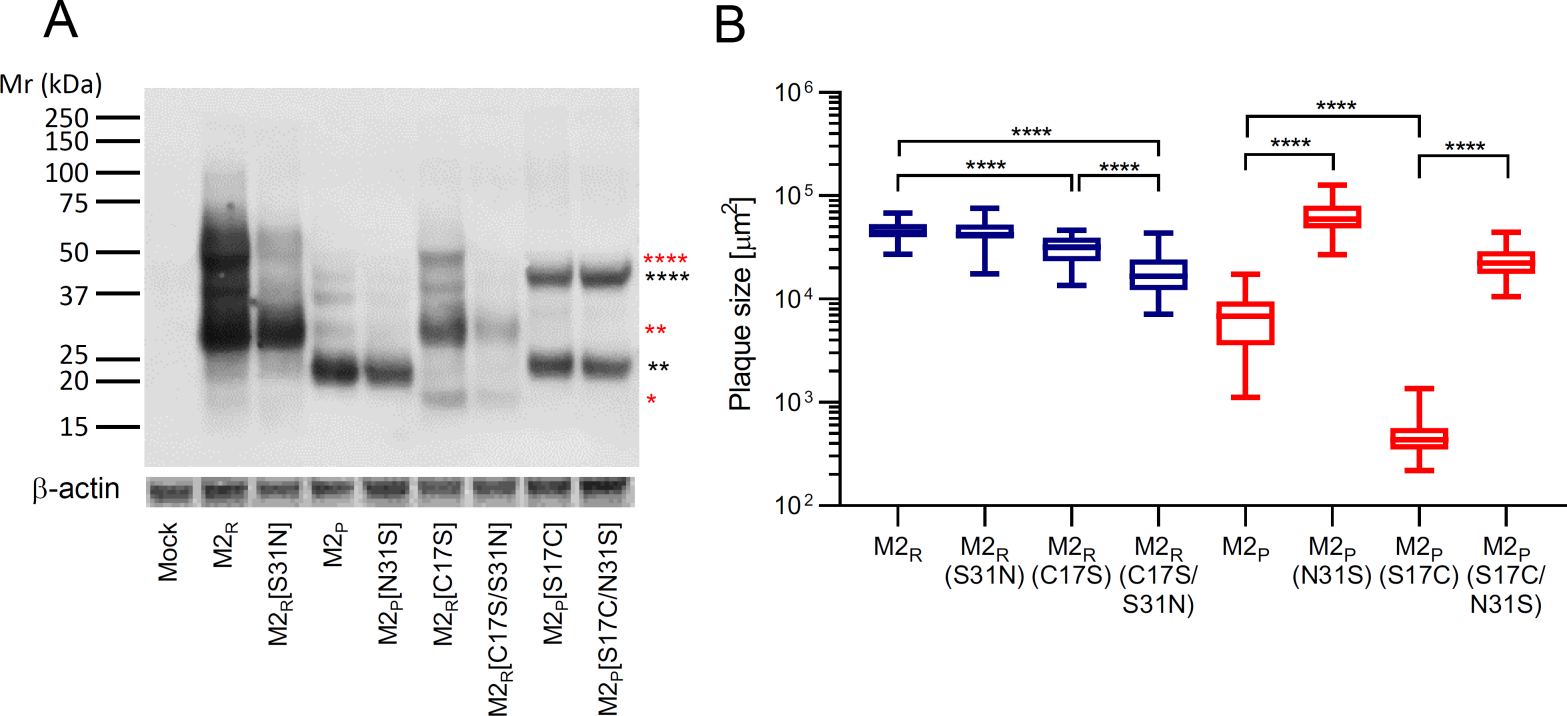
Importance of Cys17 on M2 tetramer stability and the metastable conformation of acid-sensitive HA. (**A**) BHK-21 cells were transfected with expression plasmids encoding the indicated [HA]-tagged M2 proteins. At 24 hours post-transfection, the cells were lysed and proteins separated by SDS-PAGE (4%–12% gradient gel) under non-reducing conditions. The proteins were blotted onto nitrocellulose membranes and detected using a mAb directed to the [HA] epitope and an antibody directed to β-actin as loading control. The positions of molecular weight markers are depicted, as well as the positions of the monomeric (*), dimeric (**), and tetrameric (****) M2_P_ (black stars) and M2_R_ (red stars) proteins. (**B**) Plaque size assays were performed by infecting BHK-21 cell monolayers with either VSVΔG(HA_R_:NA_R_:M2) encoding one of the indicated M2 proteins. The viruses were allowed to spread in the cell monolayer covered with methylcellulose overlay medium for 30 hours. The cells were fixed with formalin, permeabilized with TX-100, and immunolabeled using a mAb directed to the VSV M protein. Box & Whiskers (min to max) plots of at least 20 plaques per virus are depicted. Plaque sizes were compared by Brown-Forsythe and Welch ANOVA test followed by multiple comparisons tests (*P < 0.05, **P < 0.01, ***P < 0.001, and ****P < 0.0001).

As specified in Fig. 1A, the M2 proteins of H17N10 and H18N11 lack Cys17 in the ectodomain, which may result in lower stability of the M2 tetramers as only one intramolecular disulfide bond can be formed (Fig. 2C) (45). To study the impact of a second cysteine residue in the M2_P_ ectodomain on the stability of the tetrameric form of the protein, we introduced the S17C substitution into the ^[HA]^M2_P_ cDNA. Additionally, the ^[HA]^M2_P_(N31S) and ^[HA]^M2_P_(S17C/N31S) mutants were generated as the N31S substitution was linked to enhanced PCA. In addition, we destroyed cysteine 17 of ^[HA]^M2_R_ by introducing the C17S substitution, while S31N and C17S/S31N served as controls. Western blot analysis of transfected cell lysates revealed a lower abundance of stabilized tetramers M2_R_(S31N) compared to M2_R_ (Fig. 7A). A reduced proportion of stabilized tetramers and an increase in monomeric protein was observed for the mutant M2_R_(C17S), while M2_R_(C17S/S31N) appeared only as monomeric and dimeric but not as tetrameric form. The M2_P_(N31S) substitution did not lead to the appearance of M2_P_ tetramers in the non-reducing SDS-PAGE (Fig. 7A). However, an increased formation of stabilized tetramers was observed for both M2_P_(S17C) and M2_P_(S17C/N31S).

To study the impact of a cysteine 17 on M2 PCA, we generated chimeric VSVΔG(HA_R_:NA_R_:M2) encoding the corresponding wild type and mutant M2 proteins. Analysis of plaque size formation by these viruses revealed that VSVΔG[HA_R_:NA_R_:M2_P_(S17C)] induced smaller plaques than VSVΔG(HA_R_:NA_R_:M2_P_) and VSVΔG[HA_R_:NA_R_:M2_P_(N31S)] (Fig. 7B), indicating that the new cysteine at position 17 did not increase but rather reduced plaque size. In contrast, infection with VSVΔG[HA_R_:NA_R_:M2_R_(C17S)] led to reduced plaque size compared to VSVΔG(HA_R_:NA_R_:M2_R_), and this was even more reduced for VSVΔG[HA_R_:NA_R_:M2_R_(C17S/S31N)]. Thus, while M2_R_ requires Cys17 to fully preserve the metastable conformation of acid-sensitive HA, a cysteine at this position is detrimental for the corresponding activity of M2_P_.

### M2_P_ protein is rapidly internalized from the cell surface of H18N11-infected cells

**Fig 8:**
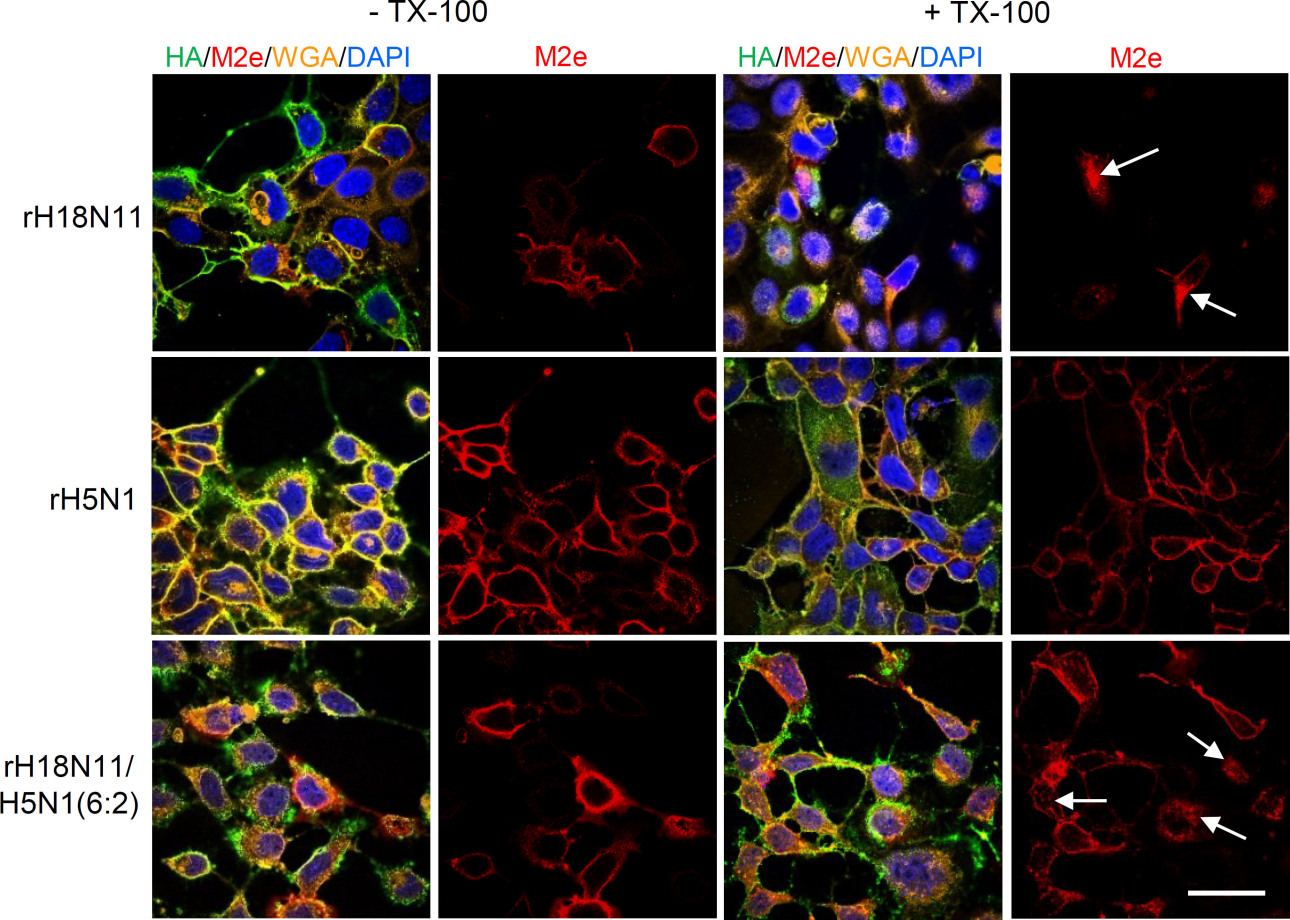
Cell surface expression and endocytosis of M2 proteins in virus-infected cells. MDCK-II cells were infected with the indicated recombinant IAV using an mo.i. of 1 ffu/cell. At 24 hours p.i., the cells were incubated for 1 hour at 37°C with the E10 antibody directed to the M2 ectodomain (M2e) along with antibodies directed to the H18 or H5 hemagglutinin. The cells were washed, fixed with formalin, and either left non-permeabilized (-TX-100) or permeabilized (+ Triton X-100). Subsequently, the cells were incubated with fluorescence-labeled secondary antibodies and WGA-rhodamine. The nuclei were stained with DAPI. Bar = 50 µm.

Analysis of transfected BHK-21 cells suggested that M2_P_ is rapidly internalized from the cell surface while M2_R_ stays at the plasma membrane (see Fig. 3C). To rule out the possibility that M2_P_ associates with other viral proteins to stay at the plasma membrane, we infected MDCK-II cells with either H18N11, H5N1 or H18N11/H5N1(6:2), a chimeric virus containing six out of the eight RNA segments of H18N11 while two modified RNA segments encoded the HA and NA proteins of H5N1. At 24 hours p.i., the live cells were incubated for 1 hour at 37°C with the E10 antibody directed to the conserved N-terminal region of M2, and with antibodies specifically recognizing either the H5 or H18 hemagglutinin. Thereafter, the cells were washed, fixed with formalin, and either permeabilized or not permeabilized with Triton X-100 prior to incubation with a fluorescent secondary antibody (Fig. 8). Although several non-permeabilized cells were stained positive for the HA antigen of H18N11, only some of these cells also showed labeling of M2_P_ by the E10 antibody. In permeabilized cells, the E10 antibody bound to intracellular vesicles, indicating that the antibody/antigen complex had been internalized during the incubation at 37°C. MDCK-II cells that had been infected with H5N1 showed a completely different phenotype: All infected (HA-positive) cells were also labeled at the plasma membrane with the E10 antibody. In permeabilized cells, the E10 staining was still confined to the cell surface indicating that the M2_Y_ (H5N1) protein stayed at the plasma membrane and was not internalized at 37°C. When the MDCK-II cells were infected with the chimeric H18N11/H5N1(6:2) virus, a large fraction of the infected cells showed M2_P_ expression at the surface. In permeabilized cells, the E10 antibody was bound to intracellular vesicles, however, a considerable fraction was still found at the cell surface, most likely because one of the H5N1 glycoproteins interfered with M2_P_ internalization (see Discussion).

### Detection of proton flux across the plasma membrane of infected cells

**Fig 9:**
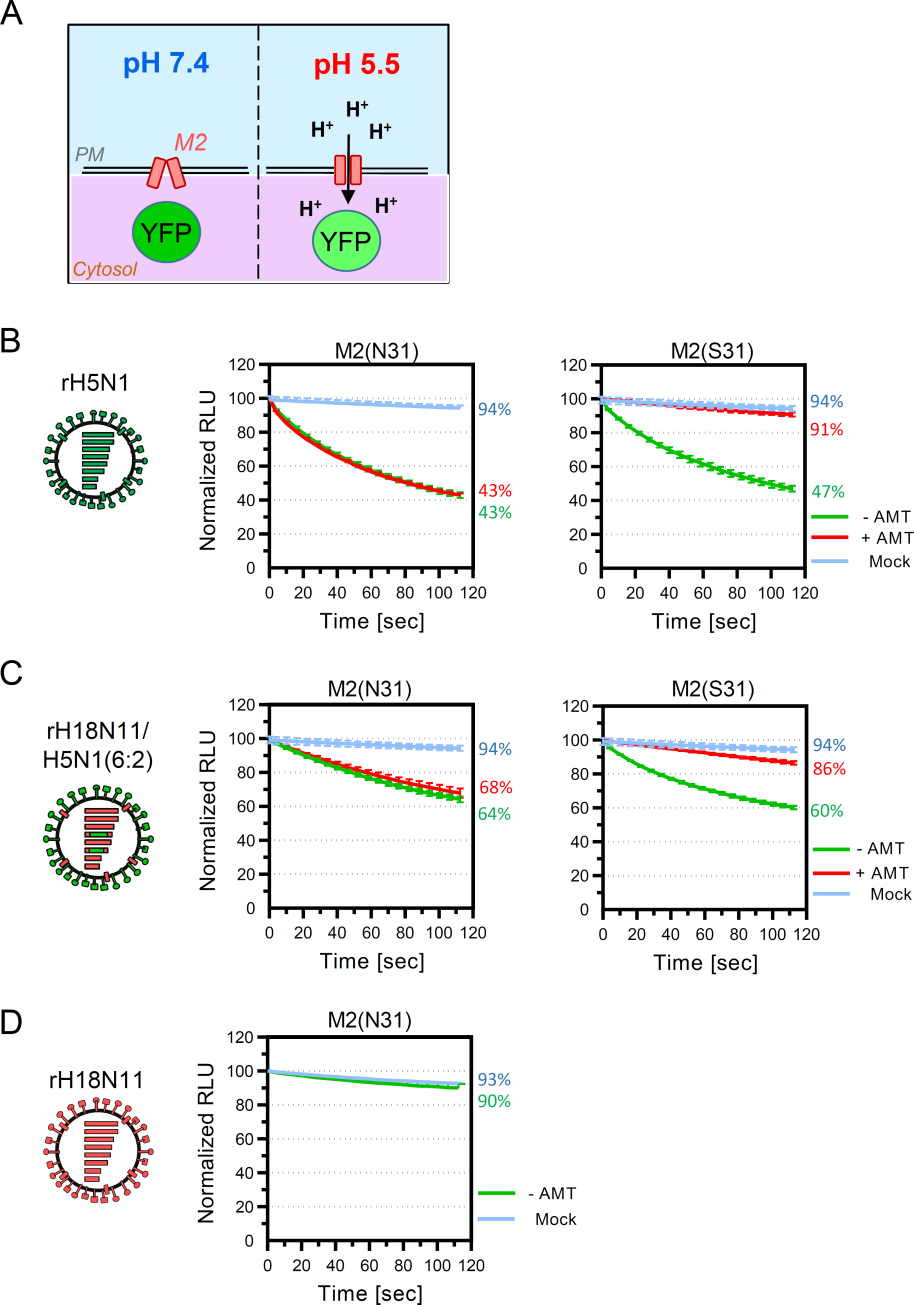
Detection of proton flux across the plasma membrane of infected cells using pH-sensitive YFP as probe. (**A**) Principle of the bioassay for measuring proton channel activity using the pH-sensitive yellow fluorescent protein (YFP). (**B–D**) RIE1495-YFP cells were either mock-infected or infected with one of the indicated wild type or mutant viruses using an m.o.i. of 2 ffu/cell. At 14 hours p.i., the cells were exposed to pH 5.5, and quenching of YFP fluorescence was recorded. (**B**) Analysis of cells infected with either rH5N1-M2(N31) (left panel) or rH5N1-M2(S31) (right panel) in the presence (red line) or absence (green line) of 10 µM of amantadine (AMT). Mock-infected cell are represented by a blue curve. (**C**) Analysis of cells infected with either rH18N11/H5N1(6:2)-M2(N31) (left hand) or rH18N11/H5N1(6:2)-M2(S31) (right panel) in the presence (red line) or absence (green line) of 10 µM of amantadine (AMT). Mock-infected cell are represented by a blue curve. (**D**) Detection of proton channel activity at the surface of cells infected with rH18N11-M2(N31). YFP intensity as detected at the start of the recording was set to 100%.

To address the question of whether cell surface M2_P_ would show PCA, we took advantage of MDCK-II cells stably expressing the pH-sensitive yellow fluorescent protein (YFP) (Fig. 9A). Mock-infected cells exhibited very little (5%) change in YFP fluorescence after 2 minutes incubation at pH 5.5 (Fig. 9B). However, MDCK-YFP cells showed a marked decline of fluorescence at 14 hours p.i. with rH5N1 if the cells were incubated with buffer adjusted to pH 5.5 (Fig. 9B). The influx of protons was not inhibited by amantadine if the cells were infected with rH5N1-M2(N31) encoding an AMT-resistant M2 protein (Fig. 9B, left hand graph). However, almost complete inhibition of YFP quenching was observed if the cells were infected with rH5N1-M2(S31) encoding the AMT-sensitive M2(N31S) mutant (Fig. 9B, right-hand panel). These findings indicate that the observed proton flux is mediated by the M2 protein and is not a consequence of virus-mediated damage of the plasma membrane. When MDCK-YFP cells were infected with rH18N11/H5N1(6:2) and incubated at pH 5.5, a progressive drop of fluorescence intensity was observed that reached 64% of the initial intensity after 2 minutes of incubation (Fig. 9C). Significant proton flux was also observed with cells that had been infected with rH18N11/H5N1(6:2)-M2(S31) and rH18N11/H5N1(6:2)-M2(N31). YFP quenching in cells infected with rH18N11/H5N1(6:2)-M2(S31) was sensitive to inhibition by AMT since this virus encoded the AMT-sensitive M2_P_(N31S) mutant (Fig. 9C, cf. left and right-hand graphs). In contrast to rH5N1 and H18N11/H5N1(6:2), infection of MDCK-YFP cells with rH18N11 wild-type virus did not result in quenching of the YFP fluorescence signal (Fig. 9D), suggesting that proton flux across the plasma membrane of rH18N11-infected cells is much lower than that of rH18N11/H5N1(6:2) infected cells.

### Uncoating of rH18N11 in MDCK-II cells is delayed

**Fig 10:**
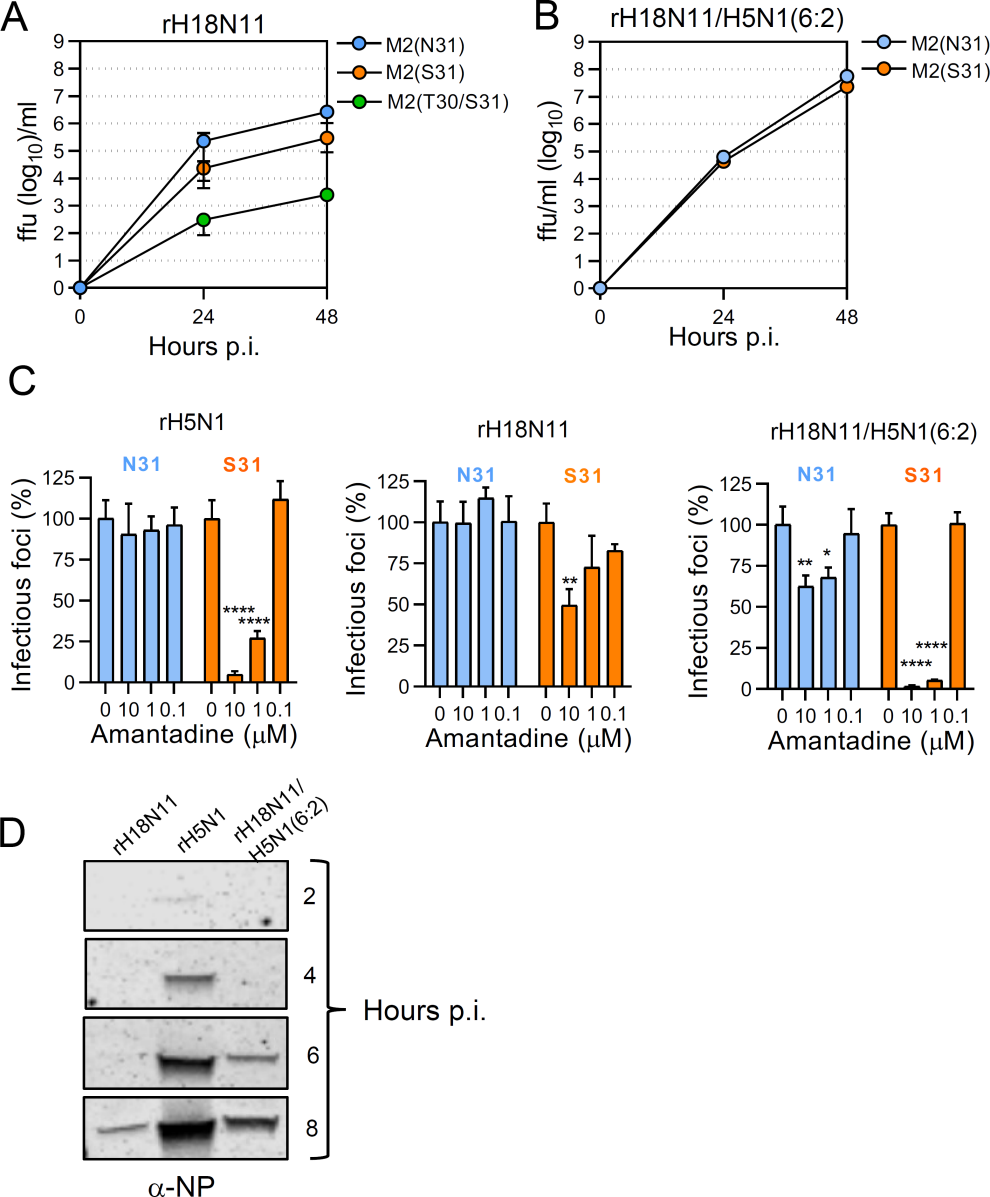
Effect of amantadine on virus uncoating. (**A**) Multistep replication kinetics of wild type and M2 mutant rH18N11 on MDCK-II cells (m.o.i. of 0.01 ffu/cell). (**B**) Impact of amantadine on virus entry as determined by plaque reduction assay. The indicated viruses were incubated with MDCK-II cells (100–200 ffu/well) in the presence of the indicated amantadine concentrations. At 14 hours p.i., the cells were fixed with formalin and permeabilized with Triton X-100. Following immunostaining of the cells using a monoclonal antibody directed to the NP antigen, the number of infectious foci was determined. The number of infectious foci detected in the absence of AMT was set to 100%. The mean and SD of three infection experiments are shown. Significant reduction of infectious foci was calculated by the one-way ANOVA test (*P < 0.05; **P < 0.01; ***P < 0.001; and ****P < 0.0001). (**C**) Relative abundance of viral proteins in purified rH18N11-M2(N31) and rH18N11-M2(S31) viruses as determined by mass spectrometry. (**D**) Entry kinetics of recombinant IAV. MDCK-II cells were infected with either rH5N1 (m.o.i. = 2 ffu/cell), rH18N11/H5N1(6:2) (m.o.i. = 2 ffu/cell), or rH18N11 (m.o.i. = 3 ffu/cell) and were lysed 2, 4, 6, and 8 hours p.i. The cell lysates were analyzed by Western blot using a monoclonal anti-NP antibody. The protein bands at 57 kDa representing NP are shown.

The PCA of classical IAV M2 proteins is known to play an important role in virus uncoating by allowing protons to enter the virus interior (24). To see whether bat IAV M2 proteins are also important for virus uncoating, we generated recombinant rH18N11 encoding either the AMT-resistant M2_P_(N31S) or the M2_P_(A30T/N31S) protein. Characterization of these viruses by multistep replication kinetics on MDCK-II cells revealed that the rH18N11-M2(N31S) replicated to ten-fold lower titers compared to wild-type rH18N11 (Fig. 10A), although this M2_P_ mutant was found to exhibit higher PCA than wild-type M2_P_ protein in the VSVΔG(HA_R_:NA_R_:M2) system (see Fig. 5D). However, the rH18N11-M2(A30T/N31S) mutant, which was unable to preserve the metastable conformation of acid-sensitive HA (see Fig. 5C), was attenuated by three log_10_ compared to wild-type rH18N11 (Fig. 10A), suggesting that PCA is important for efficient replication of this virus. Interestingly, the N31S substitution had no attenuating effect on the replication of chimeric rH18N11/H5N1(6:2) (Fig. 10B).

The role of M2 PCA in virus entry was analyzed by a plaque reduction assay using AMT as specific inhibitor of M2 PCA. For rH5N1-M2(N31), the number of infectious foci was not affected by AMT in line with the resistance of this M2 mutant to AMT (Fig. 10C, left panel). However, rH5N1-M2(S31) encoding an AMT-sensitive M2 protein, was significantly inhibited by the drug. On the contrary, AMT had only a modest inhibitory effect on plaque formation by the AMT-sensitive rH18N11-M2(S31) with 10 µM of AMT leading to only a 50% reduction of the number of infectious foci (Fig. 10C, central panel). We hypothesized that this attenuation might be due to a lower incorporation rate of the mutant M2_P_(N31S) protein into the viral envelope. However, mass spectrometry analysis of purified rH18N11-M2(S31) and rH18N11-M2(N31) particles showed that the mutant M2_P_(N31S) protein was equally well incorporated into the viral envelope as the wild-type M2_P_ protein (Fig. S3). Finally, we analyzed the effect of AMT on entry of chimeric rH18N11/H5N1(6:2). AMT had a significant inhibitory effect on the entry of the AMT-resistant rH18N11/H5N1(6:2)-M2(N31) (Fig. 10C, right panel), which might be due to a higher sensitivity of this virus to the lysosomotropic properties of AMT. However, the inhibitory effect of AMT on the rH18N11/H5N1(6:2)-M2(N31) encoding the AMT-sensitive M2_P_(S31) protein was much more pronounced, suggesting that uncoating of rH18N11/H5N1(6:2) is supported by M2 PCA.

It has previously been reported that AMT-mediated inhibition of M2 PCA results in delayed IAV uncoating and consequent later start of viral protein expression (64). We hypothesized that the low PCA of bat IAV M2 proteins would also lead to a slower virus entry process. To test this hypothesis, we infected MDCK-II with either rH5N1, rH18N11, and rH18N11/H5N1(6:2) and lysed the cells at 2, 4, 6, and 8 hours p.i.. Western blot analysis of the cell lysates showed that NP antigen could already be detected at 4 hours p.i. with rH5N1, while in cells infected with rH18N11 and rH18N11/H5N1(6:2) the NP antigen was not detected before 8 and 6 hours p.i., respectively (Fig. 10D). These findings support the idea that the low PCA of bat IAV M2 proteins may result in delayed virus uncoating.

## DISCUSSION

In the present study, we provide evidence that the M2 proteins of bat IAV H17N10 and H18N11 differ from the M2 proteins of conventional IAV in several aspects: (i) The bat IAV M2 proteins have highly divergent primary sequences compared to the highly conserved M2 protein of conventional IAV. (ii) The M2_P_ (H18N11) protein demonstrated significantly reduced capacity to protect acid-sensitive HA from premature low pH-induced conformational change and this effect was even more pronounced for M2_G_ (H17N10). (iii) The N31S substitution dramatically increased the capacity of bat IAV M2 proteins to rescue acid-sensitive HA. (iv) The M2_P_ (H18N11) protein is readily internalized from the plasma membrane. (v) The AH and CT domains of M2 were found to affect cell surface expression levels. All these special features of bat IAV M2 proteins may reflect functional differences between the bat and conventional IAV M2 proteins.

Using chimeric VSVΔG(HA_R_:NA_R_:M2), we observed that the M2 proteins of bat IAV H17N10 and H18N11 did not fully preserve the metastable conformation of the acid-sensitive HA_R_ in the secretory pathway (see Fig. 5A and B), indicating that the PCA of the bat IAV M2 proteins might be too low to sufficiently raise the pH in this compartment. In line with this finding, we previously observed that the N31S mutation was rapidly acquired when the chimeric bat IAV rH17N10/H7N7(6:2), which encoded for the HA and NA glycoproteins of A/seal/Massachusetts/1-SC35M (H7N7), was passaged in mammalian or avian cells (41). As the N31S substitution was shown in our study to dramatically enhance rescue of acid-sensitive HA by bat IAV M2 proteins (see Fig. 5C and D), this mutation was most likely selected in rH17N10/H7N7(6:2) to protect the HA (H7N7) from premature low pH-induced conformational change in the secretory pathway.

S31N is the prevalent mutation in conventional IAV M2 proteins, which confers resistance to AMT (65). The crystal structure of the drug-resistant M2(S31N) proton channel showed that the N31 side chains point directly to the center of the channel pore where they form a hydrogen-bonded network that disrupts the drug-binding site (10). Although it is unlikely that bat IAV have ever been exposed to AMT, the N31 residue is conserved in the M2 proteins of all bat IAV isolated so far and also confers resistance of bat IAV M2 proteins to AMT (41). We speculate that N31 has been maintained in bat IAV M2 proteins not because it confers resistance to AMT but rather because it keeps the PCA of these proteins low. It has recently been shown that IAV infection activates NLRP3 inflammasome through *trans*-Golgi network (TGN) dispersion and that high M2 PCA is responsible for this effect (56). In line with these previous findings, the Golgi apparatus stayed intact in M2_P_-transfected cells, whereas it appeared dispersed in cells expressing the conventional M2_R_ protein (see Fig. 3B). Thus, a low PCA may be regarded as a strategy to circumvent cytotoxic effects and inflammation that are normally associated with high proton-conducting activity (28 - 33), and this may favor a persistent infection of bats without causing disease (40).

In contrast to bat IAV M2 proteins where the N31S substitution had a dramatic protective effect on the conformation of acid-sensitive HA, the reciprocal S31N substitution did not affect the corresponding activity of M2_R_ (H7N1) (see Fig. 5C and D) and similar observations were made with the M2 proteins of A/Udorn/72 (H3N2) (9) and rH5N1 (see Fig. 9C). We speculate that the bat IAV M2 proteins adopt a completely different conformation compared to conventional M2 proteins: the channel is probably tightly closed and only partially opens upon exposure to low pH. The N31S mutation may change the conformation of the channel protein in such a way that the channel pore can more easily open upon exposure to low pH. However, structural analysis of the wild type and N31S mutant of the bat IAV M2 proteins is needed to prove this hypothesis. Surprisingly, when rH18N11/H5N1(6:2)-infected cells were analyzed for PCA, the corresponding activity of M2(N31)- and M2(S31)-encoding viruses was similarly high (see Fig. 9C). The reason for this phenomenon is not known yet, but co-clustering of M2 with HA at the plasma membrane might have had an impact on M2 protein conformation as well (66).

A well-established function of IAV M2 proteins is to preserve the metastable conformation of acid-sensitive HA in the acidic milieu of the secretory pathway. There are two features that may render an HA protein highly sensitive to low pH, and this is exemplified by the HA_R_ of A/FPV/chicken/Rostock/1934 (H7N1). First, the irreversible conformational change of HA_R_ is triggered at a relatively high pH threshold (pH ~5.9) (67). Second, HA_R_ contains a multibasic cleavage site that is cleaved by the TGN resident prohormone convertase furin (68). If this cleavage site was changed into a monobasic sequence motif, HA_R_ was not cleaved by furin, but required exogenously added trypsin for activation. The chimeric VSVΔG(HA_R_:NA_R_:GFP) encoding this HA_R_ cleavage mutant replicated as efficiently as VSVΔG(HA_R_:NA_R_:M2_R_) (see Fig. 4D), indicating that it did not rely on M2 PCA. Thus, only HA which is proteolytically processed in the secretory pathway may be at risk for a low-pH induced premature conformational change. The HA proteins of bat IAV H17N10 and H18N11 possess a monobasic cleavage site, which is recognized by the plasma membrane-resident transmembrane serine protease TMPRSS2 (69). It is not known whether the bat IAV HA proteins would also be cleaved by a bat homolog of the human airway protease (HAT) that resides in the Golgi and was shown to activate the HA of pandemic 2009 IAV (H1N1) (70). The situation of HA_P_ (H18N11) proteolytic cleavage in the Golgi can be mimicked by introducing a polybasic cleavage site into the bat IAV HA proteins (63). However, even in this context, M2 did not improve replication of chimeric VSVΔG(HA_P_(pb):NA_P_:M2_P_) when compared to the M2_P_-deficient VSVΔG(HA_P_(pb):NA_P_:GFP) (see Fig. 4F). The modified HA_G_ (H17N10) and HA_P_ (H18N11) proteins containing multibasic cleavage motifs were shown to induce membrane fusion when exposed to pH 5.4 and 5.6, respectively (63), suggesting that the bat IAV HA proteins are relatively acid-stable and are probably not at risk of a premature low-pH induced conformational change in the Golgi. A similar observation has been made with the equine IAV A/Cornell/74 (H7N7) strain. The acid-stable HA of this virus is also cleaved in the TGN but does not rely on M2 PCA to keep its metastable conformation in the secretory pathway (71). Based on these findings we conclude that bat IAV do not depend on M2 PCA to protect their HA proteins from a premature conformational change during transport through the acidic compartments of the secretory pathway.

A major function of the IAV M2 ion channel protein is to assist virus uncoating by allowing protons to enter the interior of the virion, which in turn facilitates the dissociation of the M1 protein from the inner leaflet of the viral envelope and from the ribonucleoprotein complexes (22, 54, 72). To study the impact of M2 PCA for virus uncoating, we generated recombinant IAV encoding either AMT-sensitive M2(S31) or AMT-resistant M2(N31). A plaque reduction assay showed that entry of the AMT-sensitive rH18N11/H5N1(6:2)-M2(N31S) was significantly inhibited by low concentrations of AMT (1 µM), indicating that M2_P_ PCA supported virus uncoating. Surprisingly, rH18N11-M2(S31) entry was only marginally affected by AMT, although this virus encoded for the same AMT-sensitive M2_P_(S31). We noticed that rH18N11-M2(S31) replicated to significantly lower titers compared to wild-type rH18N11-M2(N31). This attenuated phenotype was not due to impaired incorporation of M2_P_(N31S) into the viral envelope (see Fig. S3), but might be due to an overall more compromised budding efficacy of the mutant virus. Interestingly, the N31S substitution affected the fitness of rH18N11-M2(S31) but not that of rH18N11/H5N1(6:2)-M2(S31), suggesting that the defect caused by the N31S substitution could be somehow compensated by the envelope glycoproteins of H5N1 (see discussion below). When the N31S substitution was combined with the mutation A30T, M2_P_ lost its capacity to rescue acid-sensitive HA (see Fig. 5C), which resulted in severely compromised replication of rH18N11-M2(A30T/N31S) (see Fig. 10A), suggesting that M2 PCA is important for efficient uncoating. Nevertheless, rH18N11-M2(A30T/N31S) still replicated at low levels, in line with the previous observation that IAV can undergo multiple cycles of replication in the absence of detectable PCA (73). All these experiments point to low PCA of wild-type M2_P_ protein which might explain the delayed entry of rH18N11 and rH18N11/H5N1(6:2) (64).

We surprisingly observed that in contrast to the conventional M2_R_ (H7N1) protein, the M2_P_ (H18N11) protein predominantly localized to intracellular compartments in cells transfected with the corresponding cDNA or infected with rH18N11. Only a small fraction of M2_P_ was detected at the plasma membrane at steady state. Using chimeric M2 proteins, we found that the AH and CT domains were responsible for this low cell surface expression level and it is tempting to speculate that these domains contain signals that mediate rapid internalization of the M2 protein from the plasma membrane. In fact, the M2 proteins of both H17N10 and H18N11 contain one or two potential internalization signals that match the sequence motif Y-X-X-Φ. However, these motifs are located in the membrane-proximal part of the cytoplasmic domain and it is currently not known whether they can actually interact with the AP-2 adaptor protein that participates in clathrin-mediated endocytosis from the plasma membrane (74). In addition, the bat IAV M2 proteins contain several lysine residues in the distal part of their CT domains (see Fig. 1A) that might be targeted by the E3 ubiquitin ligase MARCH8 for ubiquitination-dependent endocytosis and degradation (75).

Bat IAV (H18N11) replicated to only low titers on MDCK-II cells (see Fig. 10A). As IAV M2 proteins are major players in virus assembly and budding (51, 52, 76 - 78), the low-level cell surface expression of the M2_P_ protein might be at least partially responsible for the low numbers of infectious particles produced. We found that the cell surface expression levels of M2_P_ were significantly enhanced in cells infected with rH18N11/H5N1(6:2), suggesting that the H5N1 envelope glycoproteins were able to prevent internalization and degradation of M2_P_ (Fig. 8). Interestingly, treatment of cells with sialidase has been shown to selectively inhibit caveolar endocytosis (79), while the cellular membrane-bound sialidase NEU3 has been shown to inhibit clathrin-mediated endocytosis (80). Thus, it might be that the sialidase activity of the H5N1 NA protein is responsible for the M2_P_ internalization block. M2_P_ protein that accumulates at the plasma membrane might assist in virus budding and release more efficiently than M2 protein that is internalized. If this idea holds true, it would explain why chimeric rH18N11/H5N1(6:2) replicated to higher titers than rH18N11 which is devoid of sialidase activity (35, 81). It would also explain why infection of MDCK-II cells with H18N11 in the presence of exogenous sialidase resulted in higher virus titers (39).

In summary, our experimental work using acid-sensitive HA and YFP in combination with the specific M2 proton channel inhibitor amantadine suggest that the M2 proteins of H17N10 and H18N11 bat IAV have unusually low PCA, which might represent a strategy by which toxic effects that are associated with high PCA such us triggering the inflammasome or disturbing cellular ion homeostasis are avoided (31 - 33). Furthermore, the rapid internalization of M2 from the plasma membrane and the lack of sialidase activity which could interfere with M2 internalization might represent a viral strategy to evade the host immune responses by keeping the levels of viral particles and the amount of viral antigens that are presented to the immune system low. All this may favor chronic or persistent infections in bats.

## MATERIALS AND METHODS

### Cells

BHK-21 cells were obtained from the German cell culture collection (DSZM, Braunschweig, Germany) and grown in Glasgow`s minimal essential medium [GMEM, (Life Technologies, Zug, Switzerland)] supplemented with 5% fetal bovine serum (FBS) (FBS Gold, PAN BIOTECH, cat. no. P30-3033). The transgenic BHK-21 cell clone BHK-G43 expressing the vesicular stomatitis virus G protein in a regulated manner was maintained as described previously (64). I1-hybridoma (ATCC Manassas, VA, USA, CRL-2700) secreting an IgG2a neutralizing antibody directed to the VSV glycoprotein G (82), and HB65-hybridoma (ATCC, HB-65) secreting an IgG2a antibody against NP were maintained in minimal essential medium (MEM, Life Technologies) supplemented with 10% FBS. The 23H12-hybridoma secreting anti-VSV-M IgG2a antibody was purchased from KeraFAST, Boston, MA, USA). Human embryo kidney (HEK) 293T cells (ATCC, CRL-3216) were cultured using DMEM with 10% FBS. The canine cell lines MDCK-II and RIE-1495, which are both susceptible to infection with bat IAV (65), were cultured with MEM and 5% FBS.

### Production of immune sera

Immune serum directed to the HA antigen of A/chicken/Yamaguchi/7/2004 (H5N1) has been produced in a previous study by immunization of chickens with VSV*ΔG(HA_H5_) vector vaccine (83). For production of immune serum directed against the H18N11 HA antigen, 7-week-old New Zealand white rabbits (Charles River, Lyon, France) were immunized via the intramuscular route with 10^8^  f.f.u. of VSV*ΔG-H18pb, a recombinant VSV vector encoding the HA antigen of A/flat-faced bat/Peru/033/2010 (H18N11) (63). Four weeks after the primary immunization, the animals were immunized a second time using the same vector vaccine, route, and dosage. Three weeks after the second immunization, the rabbits were bled under anesthesia. Sera were prepared by centrifugation of the coagulated blood and stored in aliquots at −20°C. Animal immunization experiments were performed in compliance with the Swiss animal protection law and approved by the animal welfare committee of the Canton of Bern (authorization number BE-128/19).

### Construction of recombinant plasmids and site-specific mutagenesis

The following M2 genes were synthesized by GenScript Biotech Corporation (Piscataway, NJ, USA): M2_R_ gene of A/FPV/chicken/Rostock/1934 (H7N1) (GenBank acc. no. M55475), M2_Y_ gene of A/chicken/Yamaguchi/7/2004 (H5N1) (GenBank acc. no. GU186709), M2_E_ gene of A/bat/Egypt/381OP/2017 (H9N2) (GenBank acc. no. MH376903), M2_G_ gene A/little yellow-shouldered bat/Guatemala/164/2009 (H17N10) (GenBank acc. no. CY103887), and the M2_P_ gene of A/flat-faced bat/Peru/033/2010 (H18N11) (GenBank acc. no. CY125948). The cDNA was amplified by PCR and ligated into the *Nhe*I site of the pCDNA6/V5-HisB plasmid (Life Technologies, cat. No. V220-01). By using a forward primer with an elongated 5′ end that encoded for a new start codon followed by the [HA]-epitope YPYDVPDYA, the M2 proteins were tagged at the N-terminus, so they could be all detected with same monoclonal antibody. Chimeric M2 proteins which combined structural domains of M2_R_ and M2_P_ were generated by swapping cDNA fragments encoding the ectodomain (aa 1-25), transmembrane domain (aa 26-44), amphipathic helix (aa 45-62), and cytoplasmic tail (63-96/97) using an overlapping PCR technology (84). Site-directed mutagenesis was performed to generate the following mutants: M2_R_(S31N), M2_R_(C17S), M2_R_(C17S/S31N), M2_P_(N31S), M2_P_(S17C), M2_P_(S17C/N31S), M2_P_(A30T/N31S), and M2_G_(N31S). The sequences of all mutant and chimeric M2 genes were verified by Sanger sequencing.

For generation of chimeric VSV (serotype Indiana, GenBank acc. no. J02428) encoding for all three IAV envelope glycoproteins, the plasmid pVSVΔG(HA:GFP) encoding an engineered VSV genome with six transcription units (85) was treated with *Mlu*I and *Nhe*I endonuclease restriction sites, resulting in the deletion of the HA and GFP genes and the intergenic region between them. A cassette comprising the VSV transcription units four to six with the open reading frames replaced by the endonuclease restriction sites *Mlu*I, *Xho*I, and *Nhe*I, respectively, was synthesized by Genscript (Piscataway, NJ, USA) and inserted into the endonuclease-treated and gel-purified pVSVΔG(HA:GFP) plasmid. The *Mlu*I and *Xho*I sites of the resulting plasmid were then filled with the HA and NA genes of A/FPV/chicken/1934 (H7N1) (GenBank acc. nos. M24457  and X52226) while the *Nhe*I site was filled with the GFP gene (GenBank acc. no. U55762) resulting in the recombinant plasmid pVSVΔG(HA_R_:NA_R_:GFP). The GFP gene in the sixth transcription unit was then replaced with either the HIV-1 vpu gene (GenBank acc. no. NC_001802) or one of the following M2 genes: M2_R_ gene of A/FPV/chicken/Rostock/1934 (H7N1) (GenBank acc. no. M55475), M2_Y_ gene of A/chicken/Yamaguchi/7/2004 (H5N1) (GenBank acc. no. GU186709), M2_E_ gene of A/bat/Egypt/381OP/2017 (H9N2) (GenBank acc. no. MH376903), M2_G_ gene A/little yellow-shouldered bat/Guatemala/164/2009 (H17N10) (GenBank acc. no. CY103887), M2_P_ gene A/flat-faced bat/Peru/033/2010 (H18N11) (GenBank acc. no. CY125948), BM2 gene of influenza B virus (GenBank acc. no. DQ792900.1). Furthermore, the mutant M2 proteins M2_R_(S31N), M2_R_(C17S), M2_R_(C17S/S31N), M2_P_(N31S), M2_P_(S17C), M2_P_(S17C/N31S), and M2_P_(A30T/N31S), M2_G_(N31S), as well as several chimeric M2 proteins combining domains of M2_R_ and M2_P_ were used to generate various recombinant pVSVΔG(HA_R_:NA_R_:M2) plasmids.

To study the role of proteolytic activation in priming of HA_R_ for low-pH induced conformational change, the polybasic proteolytic cleavage site EPSKK**R**K**KR**↓GLF (furin consensus sequence in bold letters) was changed into PEIPKG**R**↓GLF resulting in the HA_R_(mb) gene which was then used to replace the HA_R_ gene in pVSVΔG(HA_R_:NA_R_:GFP) and pVSVΔG(HA_R_:NA_R_:M2_R_). To study the role of the M2_P_ (H18N11) protein for preservation of the corresponding HA_P_ protein, the recombinant plasmids pVSVΔG(HA_P_:NA_P_:GFP) and pVSVΔG(HA_P_:NA_P_:M2_P_) were generated by replacing the corresponding genes in pVSVΔG(HA_R_:NA_R_:GFP) by those from A/flat-faced bat/Peru/033/2010. In addition, the plasmids pVSVΔG(HA_P_(pb):NA_P_:GFP) and pVSVΔG(HA_P_(pb):NA_P_:M2_P_) were constructed by changing the HA_P_ proteolytic cleavage site NPIKET**R**↓GLF into NPQRR**R**K**KR**↓GLF which matches the polybasic consensus sequence recognized by the cellular protease furin.

The pHW2000 plasmids encoding the eight genomic segments of A/chicken/Yamaguchi/7/2004 (H5N1) (GenBank accession nos.: AB166859-AB166866) were kindly provided by Y. Sakoda, Sapporo, Japan (86). To allow handling of this highly pathogenic avian IAV under biosafety level 2 conditions, the pHW2000-HA plasmid was modified using site-specific mutagenesis primers to change the pb proteolytic cleavage site of HA PQRER**R**K**RR**↓GLF (furin consensus sequence motif in bold letters) into the mb cleavage site PQRET**R**↓GLFG frequently found in the HA of low-pathogenic H5Nx isolates (87, 88). For generation of an amantadine-resistant H5N1 virus, the M2(S31N) mutation was introduced into the pHW2000-M plasmid encoding segment 7. For generation of chimeric rH18N11/H5N1(6:2) viruses, the pHW2000-HA(mb) plasmid (encoding the HA of A/chicken/Yamaguchi/7/2004 with a monobasic cleavage site) was modified by replacing the 3′ and 5′ non-coding regions (NCR) of this segment by nucleotides 1–131 and 1,606–1,771, respectively, of A/flat-faced bat/Peru/033/2010 (H18N11) genomic segment 4 (GenBank acc. no.: CY125945). In addition, the pHW2000-NA plasmid encoding A/chicken/Yamaguchi/7/2004 genomic segment 6 (GenBank accession no.: AB166864) was modified by replacing the non-coding regions by nucleotides 1–122 and 1,268–1,426 of A/flat-faced bat/Peru/033/2010 (H18N11) genomic segment 6 (GenBank acc. no.: CY125947).

The pHW2000 plasmids encoding the eight genomic segments of A/flat-faced bat/Peru/033/2010 (H18N11) (GenBank accession nos.: CY125942-CY125949) have been generated in a previous study (63). The substitutions M2(N31S) and M2(A30T/N31S) were introduced into the pHW2000-M plasmid encoding H18N11 genomic segment 7. For generation of chimeric rH18N11/H5N1(6:2) viruses, the pHW2000 plasmids containing segment 4 encoding the HA cleavage mutant and segment 6 encoding the NA protein of A/chicken/Yamaguchi/7/2004 (H5N1) were used along with the pHW2000 plasmids containing segments 1, 2, 3, 5, 7, and 8 of A/flat-faced bat/Peru/033/2010 (H18N11).

### Generation of recombinant VSV

The generation of G-deficient recombinant VSV has been performed in principle as described in a previous report (85, 89) Briefly, BHK-G43 cells were inoculated for 1 hour with modified vaccinia virus Ankara encoding the T7 phage RNA polymerase (MVA-T7) (90) using a multiplicity of infection of 3 f.f.u./cell. Subsequently, the cell culture medium was replaced with GMEM containing 5% FBS and 10^−9^ M mifepristone (Merck KGaA, Darmstadt, Germany), and the cells transfected with the Lipofectamine 2000 transfection reagent (Life Technologies) and a mixture of four plasmids: pVSVΔG(HA:NA:M2) [or alternatively pVSVΔG(HA:NA:GFP)] and plasmids encoding the VSV N, P, and L genes, respectively, all under the control of the T7 promoter (Kerafast, Boston, USA; cat. no. EH1012). One day post-transfection, the cells were dissociated with the help of 0.025% trypsin/EDTA (Life Technologies) and co-cultured for 24 hours with an equal number of non-transfected BHK-G43 cells in the presence of 10^−9^ M mifepristone. The following day, the cell culture supernatant was collected and clarified by centrifugation (1,200 × *g*, 10 minutes, 4°C). The clarified cell culture supernatant was then passed through a 0.2 µM pore size filter to deplete the MVA-T7. Infectious viruses passing the filter were propagated twice on mifepristone-treated BHK-G43 cells and stored in aliquots at −70°C in the presence of 10% FBS.

Infectious virus titers were determined on BHK-21 cells grown in 96-well microtiter plates. The cells were inoculated in duplicate with 40 µL per well of serial 10-fold virus dilutions for 1 hour at 37°C. Thereafter, the cells received 160 µL per well of GMEM containing 1% methylcellulose, incubated for 24 hours at 37°C, and fixed for 30 minutes with 3.7% formalin in PBS. The number of infectious foci produced by VSVΔG(HA:NA:GFP) was determined under the fluorescence microscope taking advantage of the GFP reporter. For detection of infectious foci produced by VSVΔG(HA:NA:M2), cells were permeabilized with 0.25% (vol/vol) of Triton X-100 and subsequently incubated for 60 minutes with a monoclonal antibody directed to the VSV matrix protein (mAb 23H12, diluted 1:25 with PBS, KeraFast, Boston, MA, cat. no. EB0011) and subsequently for 60 minutes with goat anti-mouse IgG conjugated with Alexa Fluor-488 (diluted 1:500 in PBS; Life Technologies, cat. no. A28175). Infected cells were detected by fluorescence microscopy (Observer.Z1 microscope, Zeiss, Feldbach, Switzerland), and infectious virus titers were calculated and expressed as f.f.u./mL.

### Generation of recombinant IAV

Recombinant IAV were generated by transfection of co-cultured HEK-293T and MDCK-II cells with 8 pHW2000 plasmids encoding all eight viral genomic segments and Lipofectamin 2000 (Life Technologies) as transfection reagent (91). At 24 hours p.t., the cells were washed with PBS and maintained in FBS-free medium supplemented with 0.2% (wt/vol) of bovine serum albumin, 1% (vol/vol) of penicillin/streptomycin solution (Life Technologies, cat. no. 15140122), and 1 µg/mL of acetylated trypsin (Merck KGaA, cat. no. T6763). At 6 days p.t., the supernatant of the transfected cells was supplemented with 5% FBS and cleared by low-speed centrifugation (1,200 × *g*, 10 minutes, 4°C). Infectious virus that had been released into the transfected cell culture supernatant, was passaged twice on MDCK-II or RIE-1495 cells in the presence of acetylated trypsin and 100 mU of *C. perfringens* sialidase (Merck KGaA, cat. no. N2876). At 2 days p.i., the viruses were harvested, supplemented with 5% FBS, and stored in aliquots at −70°C.

For titration of IAV, MDCK-II or RIE1495 cells were seeded into a 96-well cell culture plate (10′000 cells/well). Serial virus dilutions (10^−1^ to 10^−6^) were prepared in FBS-free MEM medium, and 40 µL of each dilution was added to the wells in duplicates. Following an incubation for 90 minutes at 37°C, 160 µL of MEM containing 1% (wt/vol) methylcellulose, 2% FBS, and 1% (vol/vol) penicillin/streptomycin solution were added to each well. At 24 hours p.i., the overlay medium was aspirated and the cells fixed for 15 minutes with 3.7% formalin in PBS. Excess formalin was quenched by washing the cells twice with PBS containing 0.1 M glycine and once with PBS. Virus-infected cells were detected by indirect immunofluorescence. For rH18N11-infected cells chicken anti-HA(H18) immune serum (1:400 in PBS) and goat anti-chicken IgG conjugated to Alexa Fluor 488 (1:400; Life Technologies, cat. no. A11039) were used. For cells infected with rH5N1 or rH18N11/H5N1(6:2), rabbit anti-HA(H5) immune serum (1:400 in PBS) and goat anti-rabbit IgG conjugated to Alexa Fluor 488 (Life Technologies, cat. no. A11034) were used for immunostaining. The number of infected cell foci was determined with an Observer.Z1 fluorescence microscope (Zeiss, Feldbach, Switzerland) using a 10× objective. Virus titers were calculated and expressed as f.f.u./mL.

To verify that any mutation introduced into the viral genome was still present after virus passaging, total RNA was either extracted from infected cell lysate (NucleoSpin RNA Mini Kit, Macherey-Nagel, Oensingen, Switzerland, cat. no. 740955) or from infectious virus stocks (NucleoSpin Virus, Macherey-Nagel). Reverse transcription (Invitrogen SuperScript III first-strand synthesis system, Life Technologies, cat. no. 10308632) was performed using the Uni-12 oligonucleotide (5′-AGCAAAAGCAGG-3′) for priming which is complementary to the 3′ end of the conserved region of the viral cRNA (92). Subsequently, viral genomic segments were amplified with segment-specific primers (92) using a high-fidelity DNA polymerase (Phusion high-fidelity DNA polymerase, Life Technologies, cat. no. F-530). Sanger DNA sequencing of the amplicons was performed using the BigDye Terminator v3.1 cycle sequencing kit (Life Technologies, cat. no. 4337458) and analyzed using a SeqStudio Genetic Analyzer System (Thermo Fisher Scientific, Bremen, Germany).

### Plaque size assay

BHK-21 cells were infected (m.o.i. of 0.01 f.f.u./cell) with either VSVΔG(HA_R_:NA_R_:GFP) encoding for the GFP reporter or VSVΔG(HA_R_:NA_R_:M2) encoding the indicated wild type, mutant or chimeric M2 protein. The GMEM medium containing 1% (wt/vol) of methylcellulose was added 60 minutes p.i., and the cells subsequently maintained for 30 hours at 37°C. The cells were fixed for 15 minutes with PBS containing 3.7% formalin, washed twice with PBS containing 0.1 M glycine, and washed once with PBS. Cells were then permeabilized for 5 minutes with 0.25% (vol/vol) Triton X-100, washed twice with PBS, and incubated for 60 minutes with a monoclonal antibody directed to the VSV matrix protein (mAb 23H12, diluted 1:25 with PBS, KeraFast, Boston, MA, cat. no. EB0011) and subsequently for 60 minutes with goat anti-mouse IgG conjugated with Alexa Fluor-488 (diluted 1:500 in PBS; Life Technologies, cat. no. A28175). The cells were washed twice with PBS and analyzed using the 20× objective of an Observer.Z1 fluorescence microscope (Zeiss). About 15 to 20 infectious foci were imaged per virus construct, and plaque size was determined by using Image J software (93).

### YFP-based assay to measure M2 proton channel activity

The cDNA encoding the yellow fluorescent protein was amplified from the pcDNA6.2/C-YFP-DEST Vector (Life Technologies, cat. no. V35720) by PCR using gene-specific forward and reverse primers harboring *Kpn*I and *Hind*III endonuclease restriction sites at the 5′ prime ends, respectively. The amplicon was treated with *Kpn*I and *Hind*III restriction endonucleases and ligated into the respective cloning sites of the pCEP4 plasmid (Life Technologies, cat. no. V04450). RIE-1495 cells were transfected with the recombinant pCEP4-YFP plasmid and selected for 14 days with 250 µg/mL of hygromycin B (Invivogen, Toulouse, France; cat. no. ant-hg-1) A cell clone expressing high levels of YFP was selected and used for subsequent ion channel studies.

RIE1495-YFP cells were seeded into 96-well cell culture plates (2 × 10^4^ cells/well, six replicates for each experiment). The cells were cultured for 24 hours at 37°C and infected with IAV using an m.o.i. of 2 f.f.u./cell. At 14 hours p.i., the cells were washed once with 200 µL/well of MES buffer, pH 7.4 (50 mM MES, 100 mM NaCl, 50 mM KCl, and 1 mM CaCl_2_), and incubated with this buffer (50 µL/well) for 5 minutes at 21°C. YFP fluorescence was recorded for 2 minutes on a GloMax Discover microplate reader (Promega, Dübendorf, Switzerland) with an excitation line at 475 nm and an emission filter at 500–550 nm. If the fluorescence signal did not change during this time by more than 5%, the plasma membrane was regarded as intact and the cells were incubated with 50 L/well of MES buffer adjusted to pH 5.5. As soon as the buffer was added, the plate was returned to the microplate reader, and YFP fluorescence was recorded for 2 minutes at 5 seconds intervals. For inhibition of M2 proton channel activity, the cells were treated for 30 minutes with 20 µM of amantadine in MEM medium which was also present in the subsequent incubations with MES buffers adjusted to pH 7.4 and pH 5.5, respectively. The relative light units (RLU) measured were normalized to the initial RLU that were recorded when MES buffer pH 5.5 was added. The mean values and standard deviations (SDs) of the normalized RLUs were calculated for the six replicate measurements.

### Flow cytometric analysis of M2 cell surface expression

BHK-21 cells were seeded at 3 × 10^5^ cells per well into 6-well plates and maintained at 37°C for 24 hours. The cells were transfected with 3 µg/well of pCDNA6-M2 plasmid using 6 µL/well of Lipofectamine 2000 reagent (Life Technologies). At 4 hours p.t., the cell culture supernatant was aspirated and replaced with fresh GMEM medium supplemented with 5% FBS. At 20 hours p.t., cells were washed with Ca^2+^/Mg^2+^-deficient PBS (PBS^-/-^) and suspended in PBS^-/-^ after placing μthem for 10 minutes at 37°C. Cells were stained with the LIVE/DEAD Fixable Violet Dead Cell Stain Kit (Life Technologies, cat. No. L34964) for 30 minutes at ambient temperature, washed with PBS, and then divided into two fractions. The first cell fraction was fixed/permeabilized using the BD Cytofix/Cytoperm Kit (Becton Dickinson, Basel, Switzerland, cat. no. 554714) and stained with a mouse monoclonal antibody directed to HA epitope (Merck KGaA, Darmstadt, Germany; cat. no. SAB272196, 1:3000). The second fraction of live, non-fixed cells was incubated for 20 minutes on ice with the HA epitope-specific mAb (1:100), washed once with PBS, and incubated for 15 minutes with goat anti-mouse IgG conjugated with AlexaFluor-647 (Life Technologies, cat. no. A-21235). The cells were washed, fixed as above, and analyzed with a FACSCanto II flow cytometer (Becton Dickinson). Flowjo v9.1 software (Treestars, Inc., Ashland, OR, USA) was used for analysis of intracellular and cell surface expression of M2 protein.

### Western blot analysis

BHK-21 cells were seeded into 6-well plates (3 × 10^5^ cells/well) and transfected with pCDNA6-^[HA]^M2 constructs as described in the previous section. At 20 hours p.t., the transfected cells were dissociated with trypsin/EDTA solution, suspended in GMEM with 5% FBS, and pelleted by low-speed centrifugation. The cell pellets were lysed for 10 minutes on ice with 100 µL of NP-40 lysis buffer [50 mM Tris/HCl, pH 7.5; 150 mM NaCl; 1% (vol/vol) Nonidet P-40, 0.5% (wt/vol) sodium deoxycholate] supplemented with cOmplete proteinase inhibitor cocktail (Merck KGaA, Darmstadt, Germany, cat. no. 4693132001). Insoluble material was removed by centrifugation (21,000 × *g*, 20 minutes, 4°C). To 100 µL of solubilized proteins in the post-centrifugation supernatant, 100 µL of twofold concentrated Laemmli sample buffer with or without 100 mM of dithiothreitol (DTT) were added and heated for 5 minutes at 95°C. The denatured proteins were loaded onto a polyacrylamide gradient (4%–12%) gel (GenScript, cat. no. M00653) and separated by SDS-PAGE at constant 100 V. After the SDS-PAGE run, the gels were transferred onto nitrocellulose membranes (Macherey Nagel, cat. no. 741291) and incubated for 1 hour with a monoclonal antibody directed to the [HA] epitope (1:2000; Merck, cat. no. SAB272196) along with a rabbit antibody directed to actin (1:1000; Merck KGaA, cat. no. A5060). The nitrocellulose membranes were washed three times (10 minutes each time) with PBS containing 0.1% Tween 20 (Applichem GmbH, Darmstadt, Germany) and incubated for 1 hour with IRDye 800CW goat anti-mouse IgG (1:5000, LI-COR Biosciences, Bad Homburg, Germany, cat. no. 926-32210) and IRDye 680RD goat anti-rabbit IgG (1:5000, LI-COR Biosciences, cat. no. 926-68071). The nitrocellulose membranes were washed with PBS containing 0.1% (vol/vol) of Tween 20 and finally analyzed using an Odyssey Infrared Imaging System (LI-COR Biosciences).

### Indirect immunofluorescence analysis

BHK-21 cells (10^5^ cells/well) were seeded into 24-well cell culture plates that had been coated with collagen (Merck KGaA, Darmstadt, Germany, cat no. 125–50). The following day, the cell culture medium was replaced with 500 µL of fresh GMEM with 5% FBS and the cells were transfected with 1 µg of pCDNA6-^[HA]^M2 plasmid and 2 µL of Lipofectamine 2000 transfection reagent according to the manufacturer’s protocol. At 16–20 hours p.t., the cells were incubated for 1 hour either at 4°C or at 37°C with a mAb directed to the [HA] epitope (1:100; Merck KGaA, cat. no. SAB272196). The cells were washed 3 times with 1 mL/well of PBS and fixed for 15 minutes at 21°C with 3.6% formalin in PBS. Subsequently, the cells were permeabilized for 5 minutes with 0.25% (vol/vol) of Triton X-100 in PBS, or were left non-permeabilized. The primary antibody bound to the ^[HA]^M2 antigen was detected by incubating the cells for 1 hour with goat anti-mouse IgG conjugated to Alexa Fluor-546 (1:500; Life Technologies, cat. no. A11030). Finally, the cells were stained for 5 minutes at 37°C with 4′,6-diamidino-2-phenylindole (DAPI, 0.1 µg/mL; Merck KGaA, cat. no. 9542) and mounted in ProLong Gold Antifade Mountant (Thermo Fisher, cat. no. P10144). Image acquisition was performed on the Nikon A1R confocal microscope (Nikon Europe B.V., Amsterdam, The Netherlands) using the 60×/1.4 NA oil objective.

Indirect immunofluorescence analysis was also performed with MDCK-II cells that had been infected with IAV (m.o.i. of 1 f.f.u./mL). For detection of cell surface M2 protein at 24 hours p.i., a mAb (clone E10) directed to the M1/M2 N-terminus was used (1:100; Merck, cat. no. MABF2165).

### Mass spectrometry

MDCK-II cells were seeded into six T-150 flasks (8 × 10^6^ cells/flask) and cultured for 24 hours at 37°C. The cells were infected with rH18N11 or rH18N11-M2(N31S) using an m.o.i. of 0.3 f.f.u./cell. At 48 hours p.i., IAV released into the cell culture supernatant was collected and purified by ultracentrifugation through a density gradient according to the protocol from Hutchinson et al. (94). Briefly, infectious cell culture supernatant was clarified by a first centrifugation step at 2,000 × *g* for 30 minutes at 4°C, and then by a second centrifugation step at 18,000 × *g* for 10 minutes and 4°C). The clarified supernatant was placed onto a 10% (vol/vol) OptiPrep density gradient medium (Merck KGaA, cat. no. D1556) cushion in NTC buffer (100 mM NaCl, 20 mM Tris-HCl, pH 7.4, 5 mM CaCl_2_) and pelleted (112,000 × *g*, 90 minutes, 4°C) using an SW-32 Ti Rotor (Beckman Coulter, Nyon, Switzerland). After resuspending the pelleted virus, it was placed onto a 10%–40% OptiPrep (wt/vol) gradient and centrifuged at 209′000 × *g* for 150 minutes at 4°C using an SW-41 Ti rotor (Beckman Coulter). The virus migrated in the gradient as a visible band which was carefully aspirated with a needle, resuspended in NTC buffer, and pelleted by ultracentrifugation. The pelleted virus was finally suspended in 150 µL of NTC buffer. Fifty microliters of purified virus were denatured in lithium dodecyl sulfate (LDS) sample buffer by heating for 10 minutes at 95°C prior to loading on a 10% polyacrylamide gel. Polyacrylamide gel electrophoresis was performed for 5 to 10 minutes until the whole sample had entered the gel. The gel was fixed for 15 minutes with 20% ethanol/ 10% acetic acid and stained overnight with QC Colloidal Coomassie (Life Technologies, cat. no. LC6025). After watering the gel for 1 hour, protein bands were excised, added to a tube with 20% ethanol, and further processed by the mass spectrometry core facility of the University of Bern. The proteins were subjected to in-gel digestion with trypsin according to the method described by Gunasekera et al. (95). Peptides were run on the LTQ-orbitrap XL Mass Spectrometer (Thermo Fisher Scientific) and data interpretation was performed with MaxQuant v2.3.1.0 using the published protein sequences of A/flat-faced bat/Peru/033/2010 (H18N11) as reference (GenBank accession nos.: CY125942-CY125949).

### Statistical analysis

Data were represented as the mean ± SD. Statistical analysis was performed using the GraphPad Prism program package v8. The statistical tests used to evaluate the data are indicated in the respective figure legends.

## Data Availability

All data generated or analyzed during this study are included in this published article and its online supplemental material.

## SUPPLEMENTAL MATERIAL

Supplemental figures jvi.00388-23-s0001.pdf Figures S1 to S3.

ASM does not own the copyrights to Supplemental Material that may be linked to, or accessed through, an article. The authors have granted ASM a non-exclusive, world-wide license to publish the Supplemental Material files. Please contact the corresponding author directly for reuse.
