# Supplementary material for: The M2 proteins of bat influenza A viruses reveal atypical features compared to conventional M2 proteins
Source: J Virol. 2023 Aug 4;97(8):e00388-23. doi: 10.1128/jvi.00388-23 (PMC10506471; doi:10.1128/jvi.00388-23)
Supplement: Supplemental figures — Figures S1 to S3. [file jvi.00388-23-s0001.pdf]

Thompson *et al.*
Supplemental Material

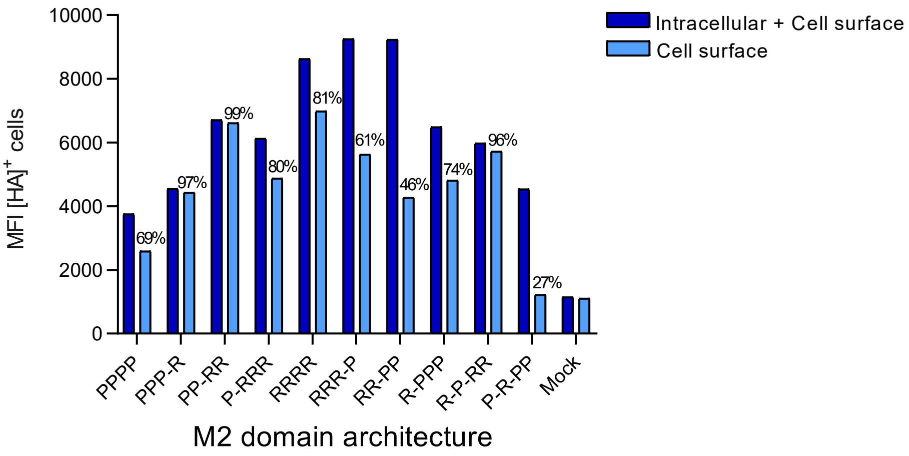

FIG S1 Cell surface and total expression levels of M2 proteins. BHK-21 cells were transfected with plasmids driving the expression of [HA]-tagged chimeric M2 proteins with the indicated domain structure. At 20 hours post transfection, the live cells were stained at 4°C with the [HA] epitope-specific mAb and subsequently fixed with formalin. One half of the transfected cell population was left non-permeabilized (detection of M2 at the cell surface) while the other half was permeabilized with Triton X-100 (total M2 labelling). All cells were subsequently incubated with the [HA]-specific mAb followed by incubation with anti-mouse IgG conjugated with AlexaFluor 647. The labelled cells were analysed by flow cytometry. The median fluorescence intensity (MFI) of [HA]-positive cells following cell surface labelling (light blue bars) and cell surface + intracellular labelling (dark blue bars) is shown. The relative M2 cell surface expression ratio is indicated.

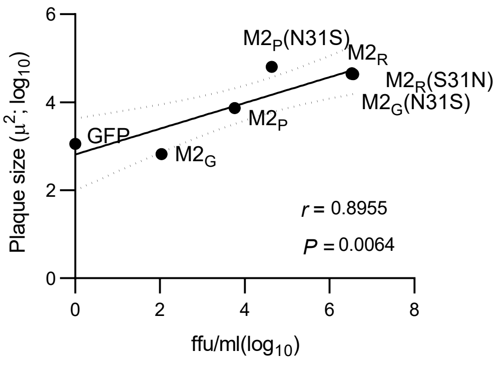

FIG S2 Linear regression analysis of plaque size versus virus titer (ffu/ml). Line and band indicate the slope of linear regression and the respective confidence intervals. r = Pearson correlation coefficient.

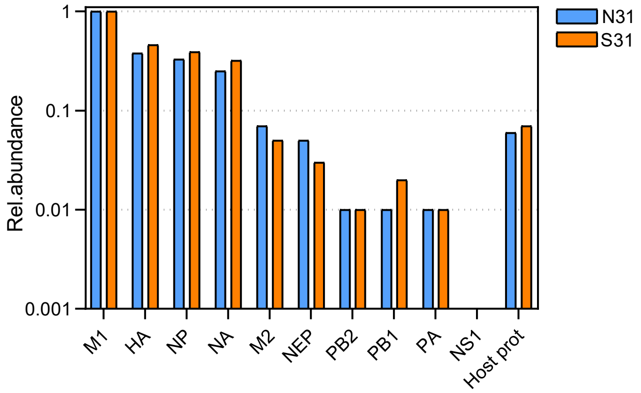

FIG S3 Incorporation of $M2_P$ protein into the H18N11 envelope. The relative abundance of viral proteins in purified rH18N11-M2(N31) and rH18N11-M2(S31) viruses was determined by mass spectrometry.
